# Astrocytic LMP2 Coordinates NF‐κB and TGF‐β1/Smad3 Signaling to Drive Neuroinflammation after Cerebral Ischemia/Reperfusion

**DOI:** 10.1002/advs.202523902

**Published:** 2026-07-27

**Authors:** Yanguang Mao, Rulan Ma, Zejing Lin, Huiying Zhao, Yuexian Liu, Yonghe Su, Xu Zhang, Xingyong Chen

**Affiliations:** ^1^ Department of Neurology Shengli Clinical Medical College of Fujian Medical University Fuzhou University Affiliated Provincial Hospital Fuzhou China

**Keywords:** astrocyte, immunoproteasome, ischemia/reperfusion injury, neuroinflammation, nuclear factor kappa‐B

## Abstract

Astrocyte reactivity critically shapes neuroinflammatory outcomes after ischemic stroke, yet the upstream regulators governing astrocyte state transitions remain incompletely defined. Here, we identify the immunoproteasome subunit low molecular weight protein 2 (LMP2) as an important modulator of astrocyte functional remodeling following cerebral ischemia/reperfusion (I/R). Using global and astrocyte‐specific knockout models, we demonstrate that LMP2 deficiency markedly reduces infarct volume, attenuates neuroinflammation, and improves neurological and cognitive outcomes. Mechanistically, LMP2 coordinately modulates inflammatory and reparative signaling networks by promoting nuclear factor kappa‐B (NF‐κB)‐dependent inflammatory activation while constraining transforming growth factor‐β1(TGF‐β1)/SMAD family member 3 (Smad3)‐associated reparative responses, thereby biasing astrocyte reactive states toward more inflammatory and maladaptive programs along the inflammatory‐reparative continuum. Conversely, LMP2 inhibition promoted more adaptive and neuroprotective astrocyte‐associated programs, enhanced neurotrophic support, and limited apoptosis under ischemic stress. Integrative transcriptomic and single‐cell analyses further revealed that astrocyte responses exist along a continuum of functional states, with LMP2 influencing the distribution of astrocyte states rather than acting as a binary switch. Collectively, these findings uncover a previously unrecognized immunoproteasome‐astrocyte regulatory axis involved in neuroinflammatory remodeling and highlight LMP2 as a promising target for precision modulation of post‐ischemic brain injury.

**Abbreviations**
BDNF: brain‐derived neurotrophic factor
CNS: central nervous system
GDNF: glial cell line‐derived neurotrophic factor
IL‐10: interleukin‐10
IL‐1β: interleukin‐1β
LMP2: low molecular mass peptide 2
MCAO/R: middle cerebral artery and reperfusion
MECL‐1: multicatalytic endopeptidase complex‐like 1
MMP9: matrix metalloproteinases‐9
NF‐κB: nuclear factor kappa‐B
OGD/R: Oxygen–glucose deprivation and reoxygenation
TGF‐β: transforming growth factor‐β
TNF‐α: Tumor necrosis factor‐α
UPS: ubiquitin proteasome system

## 1 Introduction

Ischemic stroke remains a leading cause of mortality and long‐term disability worldwide, with therapeutic interventions primarily limited to thrombolysis and mechanical thrombectomy within narrow time windows [1]. Despite advances in reperfusion strategies, more than 60% of patients continue to experience persistent neurological deficits, largely attributed to secondary neuroinflammatory cascades that exacerbate initial ischemic injury [2]. While neuroinflammation plays a critical role in determining stroke progression and outcomes, current neuroprotective approaches targeting these inflammatory processes have shown limited clinical efficacy [3]. This therapeutic gap has prompted increased interest in immune‐based interventions as potential strategies for reducing post‐stroke brain injury [4].

Within the complex cellular network of the central nervous system (CNS), astrocytes have emerged as pivotal regulators of neuroinflammation, exhibiting dual and context‐dependent functions that can either promote injury or facilitate repair [5, 6]. Following ischemic insult, astrocytes undergo rapid activation and extensive transcriptional reprogramming, transitioning through diverse reactive states. These reactive phenotypes span a continuum from pro‐inflammatory and neurotoxic programs to reparative and neurotrophic responses, collectively influencing neuronal survival, synaptic plasticity, and functional recovery [7, 8, 9, 10]. Recent single‐cell transcriptomic analyses have revealed that reactive astrocytes exist along a dynamic spectrum of activation states rather than conforming to discrete binary classifications [7], emphasizing the importance of understanding the molecular mechanisms governing these dynamic astrocyte state transitions for therapeutic development.

Current understanding of astrocyte signaling identifies nuclear factor kappa‐B (NF‐κB) as a key driver of inflammatory responses in reactive astrocytes [11], while transforming growth factor‐β (TGF‐β)/Smad signaling promotes reparative and neuroprotective programs [12]. However, the upstream molecular regulators that orchestrate the balance between these divergent signaling pathways and ultimately determine astrocyte functional outcomes remain incompletely characterized. Elucidating these regulatory mechanisms may reveal novel therapeutic targets for modulating post‐stroke neuroinflammation.

The immunoproteasome, a specialized variant of the constitutive proteasome predominantly expressed in immune and stress‐responsive cells, has gained recognition as an important modulator of inflammatory signaling across diverse pathological conditions, including neurodegenerative diseases [13, 14], autoimmune disorders [15, 16], and cerebral ischemia injury [17, 18]. Distinguished from the constitutive proteasome by its incorporation of inducible catalytic subunits, low molecular weight protein 2 (LMP2/β1i/PSMB9), LMP7 (β5i), and multicatalytic endopeptidase complex‐like 1 (MECL‐1, also known as proteasome 20S subunit beta 10 [PSMB10], β2i), the immunoproteasome is upregulated in response to pro‐inflammatory stimuli such as interferon‐γ [19]. LMP2, as a core catalytic component, serves dual functions in antigen processing for MHC class I presentation and regulation of key inflammatory signaling pathways, particularly NF‐κB activation [17, 20, 21, 22]. Although pharmacological inhibition and genetic ablation of immunoproteasome subunits have demonstrated anti‐inflammatory effects in various CNS injury models [15, 17, 23], the specific contribution of LMP2 to astrocyte functional reprogramming during cerebral ischemia/reperfusion (I/R) injury has not been systematically investigated.

Based on its established role in inflammatory signaling regulation, we hypothesized that astrocytic LMP2 contributes to ischemia‐induced neuroinflammatory injury through modulation of astrocyte reactive state transitions and their associated signaling networks. To address this hypothesis, we employed both global and astrocyte‐specific LMP2 knockout models to investigate the functional consequences of LMP2 deficiency in cerebral I/R injury. Using complementary in vivo and in vitro approaches, we assessed the impact of LMP2 deletion on infarct development, neurological recovery, astrocyte activation patterns, and inflammatory mediator expression following middle cerebral artery occlusion/reperfusion (MCAO/R). Additionally, we examined the involvement of NF‐κB and TGF‐β1/Smad3 signaling pathways as potential mechanistic mediators. Our results identify astrocytic LMP2 as a critical regulator of astrocyte reactive state transitions during cerebral ischemia, revealing novel mechanistic insights into post‐stroke neuroinflammatory regulation and establishing LMP2 as a promising therapeutic target for ischemic stroke intervention.

## 2 Materials and Methods

### 2.1 Ethical Approval and Experimental Animals

All experimental procedures were approved by the Animal Experimentation Ethics Committee of Fujian Medical University (Approval No. FJMUIACUC2020‐0059; August 12, 2020) and conducted in accordance with the National Institutes of Health Guide for the Care and Use of Laboratory Animals (eighth ed., 2011). LMP2 heterozygous type (LMP2^+/−^) Sprague‐Dawley (SD) rats were generated using CRISPR/Cas9‐mediated genome editing (Cyagen Biotechnology Co., Ltd., Guangzhou, China; License No. SCXK (Yue) 2018‐0032). LMP2^+/−^ rats were interbred to produce homozygous knockouts (LMP2^−/−^), confirmed by PCR‐based genotyping of tail DNA. In addition, astrocyte‐specific LMP2 conditional knockout C57BL/6J mice were generated by crossing floxed LMP2 mice (LMP2^flox/flox^) with GFAP‐Cre transgenic mice (Cyagen Biotechnology Co., Ltd.). Offspring genotypes were confirmed by PCR. All animals were housed in a specific pathogen‐free (SPF) facility under controlled temperature (24 ± 2°C), humidity (50 ± 10%), and a 12‐hour light/dark cycle, with ad libitum access to sterilized chow and water.

### 2.2 MCAO/R Model and Experimental Groups

Cerebral ischemia/reperfusion was established by endovascular occlusion of the right middle cerebral artery and reperfusion (MCAO/R) by withdrawal of the thread after operation 1 h as previously described in our previous publication [17]. Briefly, experiment animals aged 10–12 weeks were anesthetized with 1%sodium pentobarbital (40 mg kg^−1^; intraperitoneal injection [IP]; Sinopharm Chemical Reagent Co., Ltd., Shanghai, China). Following isolation and ligation of the right common and external carotid arteries, a nylon monofilament (Shanghai Yuyan Instruments Co., Ltd., Shanghai, China) was advanced into the internal carotid artery to occlude the MCA for 1 h, after which reperfusion was initiated by filament withdrawal. Neurological deficits were assessed by a blinded investigator using the modified Longa scoring system [24] at 2‐, 4‐, and 8‐h post‐occlusion and daily thereafter until sacrifice. Animals were randomly assigned as follows (*n* = 10∼15/group): Rats: sham and MCAO/R groups, each including LMP2^−/−^ and wild‐type (WT) littermates. Mice: sham and MCAO/R groups, each including LMP2^flox/flox^; GFAP‐Cre (abbreviation LMP2^fl/fl^; Cre^+^) (conditional knockout) and LMP2^flox/flox^ (abbreviation LMP2^fl/fl^) (control) littermates.

### 2.3 Magnetic Resonance Imaging (MRI)

Brain lesion and edema volumes were evaluated on a 7.0 T small‐animal MRI system using T2‐weighted imaging. Animals were anesthetized and secured on the MRI bed. Lesion volumes were quantified from T2‐weighted images using ImageJ (version 1.8.0) by two blinded investigators.

### 2.4 TTC Staining and Infarct Volume Measurement

At 3 days post‐MCAO/R, brains were sectioned coronally (1–2 mm thick) and incubated in 2% 2,3,5‐triphenyltetrazolium chloride (TTC; Sigma–Aldrich, St. Louis, MO, USA) for 20 min at 37°C in the darks previously described [17, 25]. Normal brain tissue exhibited red staining, while infarcted areas appeared pale. Sections were fixed in 4% paraformaldehyde (PFA), imaged, and analyzed using ImageJ (version 1.8.0). The relative infarct volume was derived by the following formula [25, 26].

$$\begin{array}{ccc} & & \mathrm{Percentage}\mathrm{hemisphereisphere}\mathrm{lesion}\mathrm{volume}\mathrm{after}\mathrm{correction}\mathrm{of}\mathrm{edema}\\ & & \;=\frac{\mathrm{total}\mathrm{infarct}\mathrm{volume}-\left(\mathrm{ipsilateral}\mathrm{hemisphere}\mathrm{volume}-\mathrm{contralateral}\mathrm{hemisphere}\mathrm{volume}\right)}{\mathrm{contraleteral}\mathrm{hemisphere}\mathrm{volume}\times 22}\times 100\% \end{array}$$

### 2.5 Hematoxylin and Eosin (H&E) and Nissl Staining

Frozen Brain Sections (10 µm) Were Prepared Using a Cryostat and Stained With Commercial H&E and Nissl Staining Kits (Beyotime Biotechnology, Shanghai, China) Following the Manufacturer's Protocols. Images Were Acquired Using a Slide Scanner (Pannoramic Midi, 3Dhistech) or Light Microscope (DMi8, Leica, Germany). The Peri‐infarct Region Was Identified on Coronal Sections as the Border Zone Adjacent to the Infarct Core. The Infarct Core Was Recognized by Overt Tissue Damage, Loss of Normal Cytoarchitecture, and Reduced Staining Intensity, Whereas Peri‐infarct Regions Were Selected From Adjacent Cortex and Striatum Where Gross Tissue Structure Was Preserved but Reactive Pathological Changes Were Evident. Quantitative Analyses Were Performed in Anatomically Matched Sections and Standardized Peri‐infarct Regions Across Animals

### 2.6 Modified Neurological Severity Score (mNSS)

Neurological function was assessed on days 3, 7, and 14 post‐MCAO/R as previously described by an investigator who blinded to the treatment and groups [25, 27]. The mNSS test includes motor, sensory, reflex, and balance assessments, with scores ranging from 0 (normal) to 18 (maximal deficit).

### 2.7 Morris Water Maze (MWM)

The Morris water maze (MWM) was performed to evaluate spatial learning and memory ability. A blindness test was conducted to exclude blind and severely dyskinetic mice on day 22 after MCAO. The four groups of mice (5–10 per group) were trained to find the platform in four trials for 5 consecutive days at days 23–27. The trial was stopped when the mice found the platform within 60 s. In case of failure, the mice were guided to the platform and left on it for 10 s to increase their memory. The time to find the platform (escape latency) and the swimming path were recorded. On day 28, the platform was removed for a probe trial, and each trial lasted 60 s. Escape latency, path length, time spent in the target quadrant, swim speed, and platform crossings were recorded and analyzed with the ANY‐maze video tracking software (Stoelting, USA) as described previously [25, 28].

### 2.8 CTX‐TNA2 Astrocytic Cell Culture

The CTX‐TNA2 rat type I astrocytic cell line, derived from the frontal cortex of 1‐day‐old rats, was purchased from Wuhan Saios Biotechnology Co., Ltd. (Product No.: CL‐016r; RRID: CVCL_3670). CTX‐TNA2 cells were cultured in complete medium consisting of high‐glucose DMEM (Beijing Solarbio Science & Technology, Beijing, China), 10% fetal bovine serum (FBS) (AusGeneX, Brisbane, Australia), and 1% antibiotic‐antimycotic (50 U mL^−1^ penicillin and 50 µg mL^−1^ streptomycin) at 37°C in a humidified incubator with 5% CO_2_.

### 2.9 Oxygen‐Glucose Deprivation and Reoxygenation (OGD/R)

The OGD/R model was performed to mimic ischemic/hypoxic/reperfusion conditions in vitro as described previously [25, 29]. The Cell Counting Kit‐8 (CCK‐8) assay (CCK‐8 Cell Proliferation and Cytotoxicity Assay Kit, Beijing Solarbio Science & Technology, Beijing, China) results indicated that the optimal OGD duration for CTX‐TNA2 cells in this study was 2 h. Briefly, astrocytes were washed with PBS (pH7.4) twice and cultured in glucose‐ and FBS‐free DMEM (Gibco). The cells were incubated in an anaerobic chamber (Changjing Biotech Co. Ltd., Changsha, China) containing a gas mixture composed of 5%CO_2_ and 95%N_2_ at 37°C for 2 h of hypoxia followed by a return to normoxic conditions with glucose‐containing DMEM supplemented with 10% FBS for 24 h reoxygenation in a humidified atmosphere containing 5%CO_2_ at 37°C. Control astrocytes were cultured in DMEM and treated similarly to those of the experimental groups.

### 2.10 LMP2‐ShRNA Lentiviral Transfection of Astrocytes

CTX‐TNA2 cells were divided into three groups: normal culture group, LMP2‐shRNA group (astrocytes transfected with LMP2‐targeting shRNA lentiviral vectors, LMP2‐konckdown group), and control‐shRNA group (astrocytes transfected with scrambled control shRNA). All three groups underwent OGD/R. Customized lentiviral vectors expressing LMP2‐targeting shRNA (GENE ID: 24967, RefSeq ID: NM_012708.2) were obtained from Zolgene Biotech, Inc., (Fuzhou, China). Lipofectamine RNAiMAX (Invitrogen, Carlsbad, CA, USA) as transfection reagent was used to transfect cells according to the manufacturer's instructions. Stable transfectants were selected with puromycin (1.5 µg mL^−1^ for 3 days, then 0.75 µg mL ^−1^ for 10 days).

### 2.11 SiRNA Transfection

As previously described [25, 29], specific siRNAs targeting NF‐κB, or TGF‐β1 were transfected into CTX‐TNA2 cells at 70∼80% confluence using Lipofectamine RNAiMAX (Invitrogen, Carlsbad, CA, USA;13778‐070) and Opti‐MEM medium (Gibco, USA) according to the manufacturer's instructions. The siRNA duplexes targeting NF‐κB(NF‐κB‐siRNA) or TGF‐β1(TGF‐β1‐siRNA) were designed and synthesized by RiboBio, Co., Ltd. (Guangzhou, China) and their sequences were as follows: NF‐κB‐siRNA: Si1: GATCAATCGCTACCAGGA, Si2: GTTCCTATAGAAGAGCAGC, Si3: GAGCATCATGAAGAAGAGT, TGF‐β1‐siRNA: Si1: GTCAACTGTGGAGCAACAC, Si2: GCACCATCCATGACATGAA, Si3: CCGCAACAACGCAATCTAT. After 48 h transfection, cells were used in subsequent experiments. The control‐siRNA‐treated astrocytes were used as a negative control.

### 2.12 Scratch Migration Assay

As described in our previous publication [29], CTX‐TNA2 cells were seeded into 6‐well plates, and the monolayer was then gently scratched with a sterile 200 µL pipette tip. Cell migration into the gap was monitored at 0, 12 and 24 h by phase contrast microscopy. Cell migration was evaluated by a widely used quantification method, which we term the area method, as previously described. Briefly, to assess migration in an indirect manner, the wound healing (WH) percentage was tracked: WH = [A(t)−A(0)]/A(0) ×100%, where A(t) is the wound area at time t and A(0) is its initial area. The area was quantified using the software ImageJ.

### 2.13 Quantitative Real‐Time PCR (RT‐qPCR)

Total RNA was extracted with TRIzol reagent (Invitrogen) from a peri‐infarct region of brains or cultured CTX‐TNA2 cells, and reverse transcribed into cDNA with Evo M‐MLV RT Mix Kit with gDNA Clean for qPCR Ver.2(Accurate Biolog, ChangSha, China). qPCR was conducted in the ABI7500 Fast Real‐Time PCR System (Applied Biosystems, CA, USA). Relative expression was calculated by the 2^˗ΔΔCT^ method, normalized to β‐actin. The primer sequences used were as follows: LMP2‐forward:CATCTACTGTGCCCTCTCGG;LMP2‐reverse: CAGCTACCATGAGATGCGCT. NF‐κB‐forward: AGAGAAGCACAGATACCACTAAGA; NF‐κB‐reverse: GTTCAGCCTCATAGAAGCCATC. LMP7‐forward: CGGGAACACCTATGCCTATG; LMP7‐reverse: GTTGACGACTCCTCCAGAATAG. MECL1‐forward: GAACGGACCTCAGCTCTACG; MECL1‐reverse: TGGAACCGGTCTTCCAACAG. PSMB6‐forward: GCAGGTGTACTCTGTTCCCA; PSMB6‐reverse: CAAAGCGAGAGCATTGGCAG. β‐actin‐forward: TCTGTGTGGATTGGTGGCTCTA; β‐actin‐reverse: CTGCTTGCTGATCCACATCTG.

### 2.14 Immunofluorescence (IF)

Animals were sacrificed with deep anesthesia and perfused intracardially with phosphate‐buffered saline (PBS) followed by ice‐cold 4%PFA. The brains were removed and post‐fixed with 4% PFA overnight at 4°C. Brains were cryoprotected by consecutive immersions in 20% and 30% sucrose in PBS at 4°C. Samples were then embedded in TissueTek OCT compound and frozen on dry ice. Brains were coronally cut into 10 µm slices. For cell IF, cells grown on coverslips in 12‐well plates were fixed with freshly prepared 4% PFA in PBS for 30 min. Samples were permeabilized with 0.3% Triton X‐100 in PBS (pH 7.4), and blocked with 10% normal goat serum (Beijing Solarbio Science & Technology, Beijing, China) at room temperature for 1 h. After the samples incubated overnight at 4°C with primary antibodies, the slices were incubated with the corresponding fluorescent secondary antibodies (Table S1). Finally, slides were mounted in antifade mountant with DAPI antifade reagent (Invitrogen) prior to imaging. Negative control sections were incubated with PBS instead of primary antibodies and showed no positive staining. Colocalization analysis was performed using the software ImageJ (Version 1.53 m). Thresholds were set to exclude background signals. At least three random fields per sample were analyzed using ImageJ software.

### 2.15 DAB‐Based Immunohistochemistry (IHC)

Paraffin‐embedded mouse brain tissues were sectioned at a thickness of 4 µm. IHC was performed according to the manufacturer's instructions (ab64261, Abcam). Briefly, paraffin sections were rinsed three times deparaffinized in xylene (2 × 10 min), and rehydrated through a graded ethanol series (100%, 95%, 85%, and 75%) to distilled water. Antigen retrieval was performed by heating sections in citrate buffer (10 mm, pH 6.0) using a microwave oven for 10–15 min, followed by natural cooling to room temperature. To block endogenous peroxidase activity, sections were incubated with 3% hydrogen peroxide (H_2_O_2_) for 10 min at room temperature. After rinsing in phosphate‐buffered saline (PBS), apply Protein Block and incubate for 10 min at room temperature to block nonspecific background staining. After washing 3 times with PBS (3 min each time), sections were then incubated overnight at 4°C with primary antibodies against C3 and S100a10 (diluted according to the manufacturer's instructions). After washing in PBS, sections were incubated with HRP‐conjugated secondary antibodies for 10 min at room temperature. Immunoreactivity was visualized using a 3,3′‐diaminobenzidine (DAB) substrate kit according to the manufacturer's protocol. Sections were counterstained with hematoxylin, dehydrated through graded ethanol, cleared in xylene, and mounted with neutral resin. Bright‐field images were acquired using identical microscope settings across all groups. For quantitative analysis, regions of interest (ROIs) were defined in the cortex and striatum, primarily focusing on peri‐infarct areas. The DAB‐positive area was quantified using ImageJ software and expressed as the percentage of positive staining area relative to the total tissue area. A uniform threshold was applied to all images within each experiment to ensure consistency. All image acquisition and quantitative analyses were performed in a blinded manner.

### 2.16 Total Protein Extraction

For total protein extraction, brain tissues or cultured CTX‐TNA2 cells or primary astrocytes were lysed in ice‐cold RIPA buffer supplemented with a complete protease inhibitor cocktail (Bimake, B142002) and phosphatase inhibitors (phosSTOP; MCE, HY‐K0021, HY‐K0022). Cell samples were washed twice with ice‐cold PBS, collected by scraping, and lysed on ice for 30 min. Tissue samples were homogenized in RIPA buffer on ice. The lysates were centrifuged at 12,000 × g for 15 min at 4°C, and the supernatants were collected as total protein extracts. Protein concentrations were determined using a BCA Protein Assay Kit.

### 2.17 Cytoplasmic and Nuclear Protein Extraction

For cytoplasmic and nuclear fractionation, CTX‐TNA2 cells cultured under normal conditions or subjected to OGD/R treatment were processed using a Nuclear and Cytoplasmic Protein Extraction Kit (Beyotime Biotechnology, Shanghai, China; Cat# P0028) according to the manufacturer's instructions. Briefly, cells were washed twice with ice‐cold PBS and harvested by gentle scraping. Cell pellets were sequentially lysed with cytoplasmic extraction buffer and nuclear extraction buffer supplemented with protease and phosphatase inhibitors. After differential centrifugation, cytoplasmic and nuclear fractions were collected separately. Protein concentrations were measured using a BCA Protein Assay Kit (Millipore, Billerica, MA, USA) according to the manufacturer's instructions.

### 2.18 Western Blotting (WB)

WB was performed as described previously [30]. Protein samples were derived from whole‐cell lysates or isolated nuclear/cytoplasmic fractions, unless otherwise specified. Equal amounts of protein (30 µg) were separated by SDS‐PAGE using Bio‐Rad electrophoresis systems (Bio‐Rad Laboratories, Hercules, CA, USA) and subsequently transferred onto 0.20 µm polyvinylidene fluoride (PVDF) membranes (Millipore, Billerica, MA, USA). Membranes were blocked with 5% nonfat milk in Tris‐buffered saline with 0.1% Tween‐20 (TBST) for 1 h at room temperature and incubated with primary antibodies overnight at 4°C (Table S2). After washing, membranes were incubated with HRP‐conjugated secondary antibodies (Table S2) for 1 h at room temperature. Protein bands were visualized using enhanced chemiluminescence (ECL) (Millipore, Billerica, MA, USA) on a Bio‐Rad imaging system (Bio‐Rad Laboratories, Hercules, CA, USA). When necessary, membranes were stripped using stripping buffer (Thermo Fisher Scientific, USA) according to the manufacturer's instructions. Band intensities were quantified using ImageJ software, and target protein expression was normalized to β‐actin or GAPDH for total/cytoplasmic proteins, and Lamin B1 for nuclear proteins.

### 2.19 RNA Sequencing Transcriptome Analysis

For transcriptomic analysis, tissue samples were consistently collected from the ipsilateral peri‐infarct cortex and basal ganglia immediately adjacent to the infarct core at 3d after MCAO/R. Visibly necrotic tissue within the ischemic core was excluded during dissection as much as possible. In this study, the peri‐infarct region was operationally defined as the cortical and basal ganglia tissue surrounding the infarct core on the ipsilateral side. In parallel, CTX‐TNA2 astrocytic cells transfected with either LMP2‐shRNA or negative control shRNA were harvested under normal culture conditions and following OGD/R treatment to mimic ischemic stress in vitro. Total RNA was extracted using standard protocols, and RNA integrity was assessed prior to library construction. Sequencing libraries were prepared using the KAPA Stranded RNA‐Seq Library Prep Kit (Illumina) and subjected to paired‐end sequencing on the Illumina NovaSeq 6000 platform. After quality control and read alignment to the reference genome, differentially expressed genes (DEGs) were identified based on fold‐change and adjusted p‐value thresholds. Subsequent Gene Ontology (GO) and Kyoto Encyclopedia of Genes and Genomes (KEGG) enrichment analyses were performed to determine the biological processes and signaling pathways associated with LMP2 modulation. Each biological replicate was processed independently for RNA extraction, library preparation, and sequencing. All transcriptome sequencing and bioinformatic analyses were conducted by Aksomics (Shanghai, China).

### 2.20 Single‐Cell RNA Sequencing and Bioinformatics Analysis

Single‐cell RNA sequencing (scRNA‐seq) was performed by Shanghai Genechem Co., Ltd. (Shanghai, China) using the 10x Genomics Chromium platform. Briefly, single‐cell suspensions were prepared from peri‐infarct cortical and striatal tissues and loaded onto the Chromium system to generate Gel Bead‐In‐Emulsions (GEMs), enabling encapsulation of individual cells with barcoded gel beads carrying unique molecular identifiers (UMIs) and cell‐specific barcodes. Reverse transcription and cDNA amplification were conducted using the Chromium Next GEM Single Cell 3′ GEM, Library & Gel Bead Kit (10x Genomics), followed by library construction according to the manufacturer's protocol. Libraries were sequenced on the Illumina NovaSeq platform (Illumina Inc., San Diego, CA, USA). Raw sequencing data were processed using the Cell Ranger pipeline (v6.1.2, 10x Genomics), including read quality control, alignment to the reference genome, UMI counting, and generation of gene expression matrices. Initial read filtering was performed using fastp (v0.21.0). Downstream analyses were conducted using Scanpy (v1.8.2) in Python. Low‐quality cells were filtered based on the following criteria: cells with fewer than 200 or more than 6000 detected genes were excluded; cells with mitochondrial gene content >10% or hemoglobin gene proportion >0.1% were removed. Genes expressed in fewer than three cells were excluded. The data were normalized and scaled, and highly variable genes (HVGs) were identified. Principal component analysis (PCA) was performed for dimensionality reduction, followed by Uniform Manifold Approximation and Projection (UMAP) for visualization. Cell clustering was performed using the Leiden algorithm with resolution parameters ranging from 0.06 to 0.5. Cell types were annotated based on canonical marker genes and reference datasets. DEGs were identified using the Wilcoxon rank‐sum test, with fold change (FC) > 2 and *p* < 0.05 considered statistically significant. GO and KEGG enrichment analyses were conducted to characterize the biological functions and pathways associated with distinct cell populations. All analyses were performed using standard pipelines implemented in Scanpy, ensuring reproducibility and consistency with widely adopted single‐cell transcriptomic workflows.

### 2.21 Statistical Analysis

Data are presented as mean ± standard deviation (SD). No data transformation or normalization was performed before statistical analysis. Data were assessed for normality prior to statistical testing. For comparisons between two groups, an unpaired two‐tailed Student's t‐test was used for normally distributed data, whereas the Mann‐Whitney U test was used for non‐normally distributed data. For comparisons among multiple groups, one‐way analysis of variance (ANOVA) followed by least significant difference (LSD) post hoc testing was used for normally distributed data, whereas the Kruskal‐Wallis test followed by Dunn's multiple‐comparison test was used for non‐normally distributed data. Repeated measurements were analyzed using repeated‐measures ANOVA followed by LSD post hoc testing, or the Friedman test when normality assumptions were not met. The sample size (n) for each experiment is indicated in the corresponding figure legends. Statistical analyses were performed using SPSS Statistics v19.0 (IBM Corp., Armonk, NY, USA) and GraphPad Prism v10.1.2 (GraphPad Software, San Diego, CA, USA). All statistical tests were two‐sided, and a *p*‐value < 0.05 was considered statistically significant.

## 3 Results

### 3.1 Effect of Complete LMP2 Gene Knockout on Cerebral Infarction Volume in Rats

**FIGURE 1:**
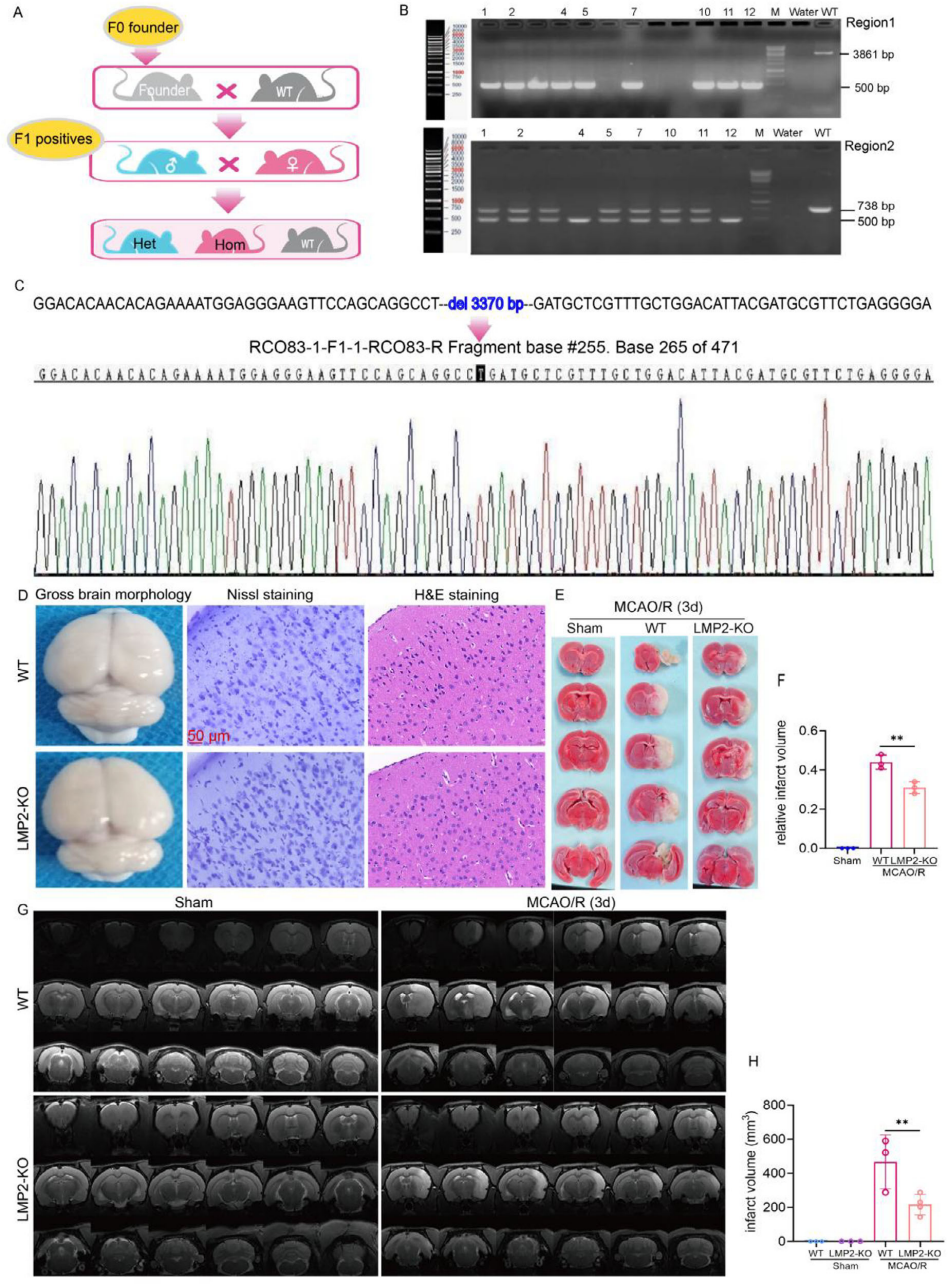
Genetic ablation of LMP2 attenuates cerebral ischemia/reperfusion injury without affecting normal brain morphology. (A) Schematic illustration of the generation of global LMP2 knockout (LMP2‐KO) rats using CRISPR/Cas9‐mediated genome editing. Het, heterozygous; Hom, homozygous; WT, wild type. (B‐C) PCR‐based genotyping (B) and Sanger sequencing (C) confirming successful deletion of the LMP2 (PSMB9) locus. Region 1 PCR: WT, 3861 bp; mutant, 500 bp. Region 2 PCR: WT, 738 bp; heterozygous, 738 bp and 500 bp; homozygous mutant, 500 bp. M, marker. (D) Representative images of gross brain morphology together with Nissl and hematoxylin‐eosin (H&E) staining demonstrating no detectable gross anatomical or histological abnormalities in LMP2‐KO rats compared with WT controls under basal conditions. *n* = 3 rats per group. Scale bar = 50 µm. (E,F) Representative TTC‐stained coronal brain sections and quantification of infarct volume 3 days after 1 h MCAO/R. White areas indicate infarcted tissue. LMP2‐KO rats exhibited significantly smaller infarct volumes than WT littermates. *n* = 3 rats per group. ^**^ *p* < 0.01. (G,H) Representative T2‐weighted magnetic resonance imaging (MRI) images and corresponding quantification of infarct volume at 3 days after 1 h MCAO/R. LMP2 deficiency markedly reduced infarct volume, while no infarction was observed in sham‐operated animals. *n* = 3 rats per group. Data are presented as mean ± SD from three independent experiments using one‐way ANOVA with LSD's post hoc test. ^**^ *p* < 0.01.

To investigate the role of LMP2 (Psmb9) in ischemic brain injury, we generated LMP2‐knockout (LMP2‐KO) rats using CRISPR/Cas9 technology and confirmed successful gene deletion by PCR and sequencing (Figure 1A–C). Under physiological conditions, no overt morphological abnormalities were observed in LMP2‐KO rat brains compared with wild‐type (WT) controls, as evidenced by gross anatomy as well as Nissl and H&E staining (Figure 1D).

To assess the impact of LMP2 deficiency on ischemic injury, TTC staining was performed in sham‐operated rats and in WT and LMP2‐KO rats subjected to 1‐hour MCAO followed by 3 days of reperfusion (MCAO/R). As expected, no infarction was detected in the sham groups, whereas prominent infarct areas were observed in MCAO/R animals at 3 days post‐reperfusion. Notably, LMP2‐KO rats exhibited significantly reduced infarct volume compared with WT controls (31.0 ± 0.3% vs. 44.0 ± 3.6%, *p* < 0.01; Figure 1E,F). To further validate these findings, T2‐weighted MRI analysis was performed 3 days after reperfusion and revealed no detectable lesions in sham animals, while MCAO/R rats showed clear hyperintense signals indicative of ischemic damage. Importantly, infarct volumes were markedly reduced in LMP2‐KO rats compared with WT counterparts (216.3 ± 60.3 mm^3^ vs. 466.6 ± 157.9 mm^3^, *p* < 0.01; Figure 1G,H). These findings demonstrate that genetic deletion of LMP2 significantly attenuates cerebral infarction following ischemia/reperfusion injury, suggesting that LMP2 plays a detrimental role in the pathogenesis of ischemic brain damage.

### 3.2 Effect of Complete LMP2 Gene Knockout on Astrocyte Activation Following Cerebral Ischemia

**FIGURE 2:**
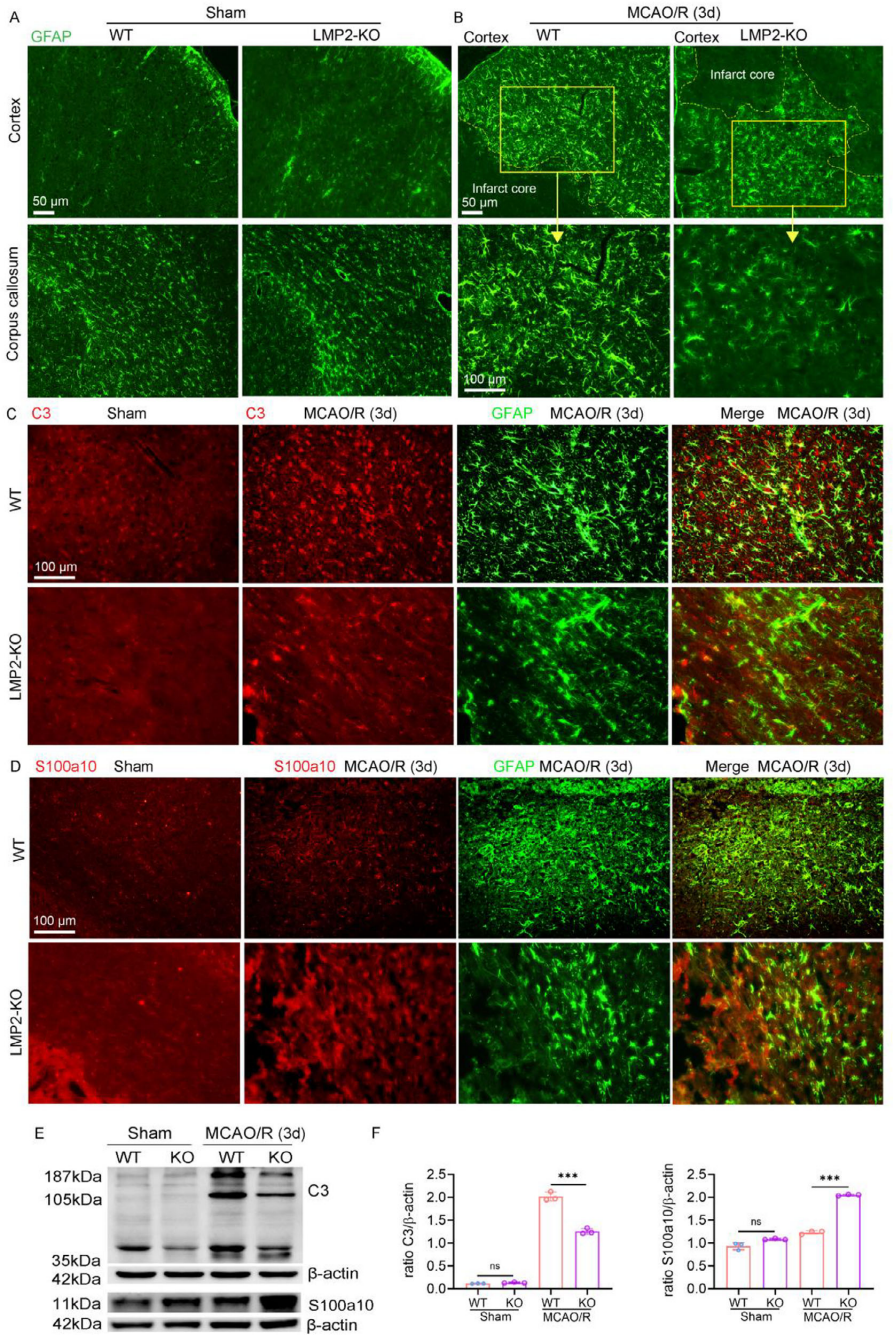
LMP2 deficiency attenuates astrocyte activation and shifts astrocyte functional states following cerebral ischemia. (A) Representative immunofluorescence images of GFAP (green) in the cortex and corpus callosum (CC) of WT and LMP2‐KO rats under sham conditions. GFAP immunoreactivity showed the expected regional distribution, with higher expression in the CC than in the cortex, and no obvious differences between genotypes under basal conditions. *n* = 3 rats per group. Scale bars = 50 µm. (B) Representative GFAP immunofluorescence images (green) in the peri‐infarct cortex of WT and LMP2‐KO rats at 3 days after 1 h MCAO/R. Astrocyte activation induced by ischemia/reperfusion, characterized by increased GFAP immunoreactivity and hypertrophic morphology, was markedly attenuated in LMP2‐KO rats. Boxed regions are shown at higher magnification below. *n* = 3 rats per group. Scale bars = 50 µm (upper panels) and 100 µm (lower panels). (C) Representative double immunofluorescence images of the pro‐inflammatory astrocyte marker C3 (red) and GFAP (green) in the peri‐infarct cortex at 3 days after MCAO/R. LMP2 deficiency markedly reduced the number of C3^+^/GFAP^+^ astrocytes compared with WT rats. *n* = 3 rats per group. Scale bars = 100 µm. (D) Representative double immunofluorescence images of the neuroprotective astrocyte marker S100a10 (red) and GFAP (green) in the peri‐infarct cortex at 3 days after MCAO/R. LMP2 deficiency enhanced S100a10^+^/GFAP^+^ astrocytes compared with WT rats. *n* = 3 rats per group. Scale bars = 100 µm. (E,F) Representative Western blots and quantitative analysis of C3 and S100a10 protein expression in the ipsilateral cortex and striatum of WT and LMP2‐KO rats under sham and MCAO/R (3d) conditions. LMP2 deficiency suppressed ischemia‐induced C3 expression while enhancing S100a10 expression. β‐actin served as the loading control. *n* = 3 rats per group. Data are presented as mean ± SD from three independent experiments using one‐way ANOVA with LSD's post hoc test. ns, not significant; ^***^ *p* < 0.001.

Given that astrocyte activation is intimately linked to infarct evolution and that interventions reducing ischemic injury often attenuate astrocyte reactivity [25, 31, 32], we investigated whether LMP2 regulates reactive astrocyte responses following cerebral ischemia. Under physiological conditions, GFAP immunoreactivity displayed characteristic regional heterogeneity, with relatively robust expression in white matter tracts such as the corpus callosum and more modest levels in cortical gray matter. Importantly, no significant differences in GFAP fluorescence intensity were detected between WT and LMP2‐KO rats under sham conditions, indicating that LMP2 deficiency does not alter baseline astrocyte architecture (Figure 2A). Following 1‐h MCAO with 3‐day reperfusion, GFAP immunoreactivity was dramatically upregulated in peri‐infarct territories, reflecting typical reactive astrogliosis. Strikingly, this ischemia‐induced astrocyte activation was significantly diminished in LMP2‐KO rats compared to WT littermates, suggesting that LMP2 promotes astrocyte reactivity during cerebral ischemia (Figure 2B).

To elucidate the functional consequences of altered astrocyte activation, we examined the expression of C3 and S100a10, established markers of neurotoxic (A1‐like) and neuroprotective (A2‐like) astrocyte phenotypes, respectively. Under sham conditions, both markers showed minimal baseline expression across all experimental groups (Figure 2C,D). At 3 days post‐MCAO/R, C3^+^ astrocytes were substantially increased in peri‐infarct regions of WT rats, indicative of robust pro‐inflammatory astrocyte activation. This neurotoxic response was markedly suppressed in LMP2‐KO animals, demonstrating reduced pathological astrocyte reactivity. Conversely, S100a10 expression was significantly elevated in peri‐infarct territories following ischemia, with LMP2‐KO rats exhibiting even greater upregulation compared to WT controls (Figure 2C,D). This pattern suggests that LMP2 deficiency promotes a phenotypic shift toward neuroprotective astrocyte programs.

Western blot analysis corroborated the immunofluorescence findings, revealing minimal basal expression of both C3 and S100a10 in sham‐operated animals, with significant upregulation observed at 3 days after MCAO/R. Crucially, LMP2‐KO rats demonstrated significantly reduced C3 protein levels concurrent with enhanced S100a10 expression compared to WT counterparts (*p* < 0.001; Figure 2E,F). Overall, these results demonstrate that LMP2 deficiency not only attenuates astrocyte activation but also shifts astrocyte functional states following cerebral ischemia, suppressing pro‐inflammatory responses while promoting reparative and neuroprotective programs.

### 3.3 RNA‐seq Analysis Reveals LMP2‐Dependent Transcriptional Reprogramming Following Cerebral Ischemia

**FIGURE 3:**
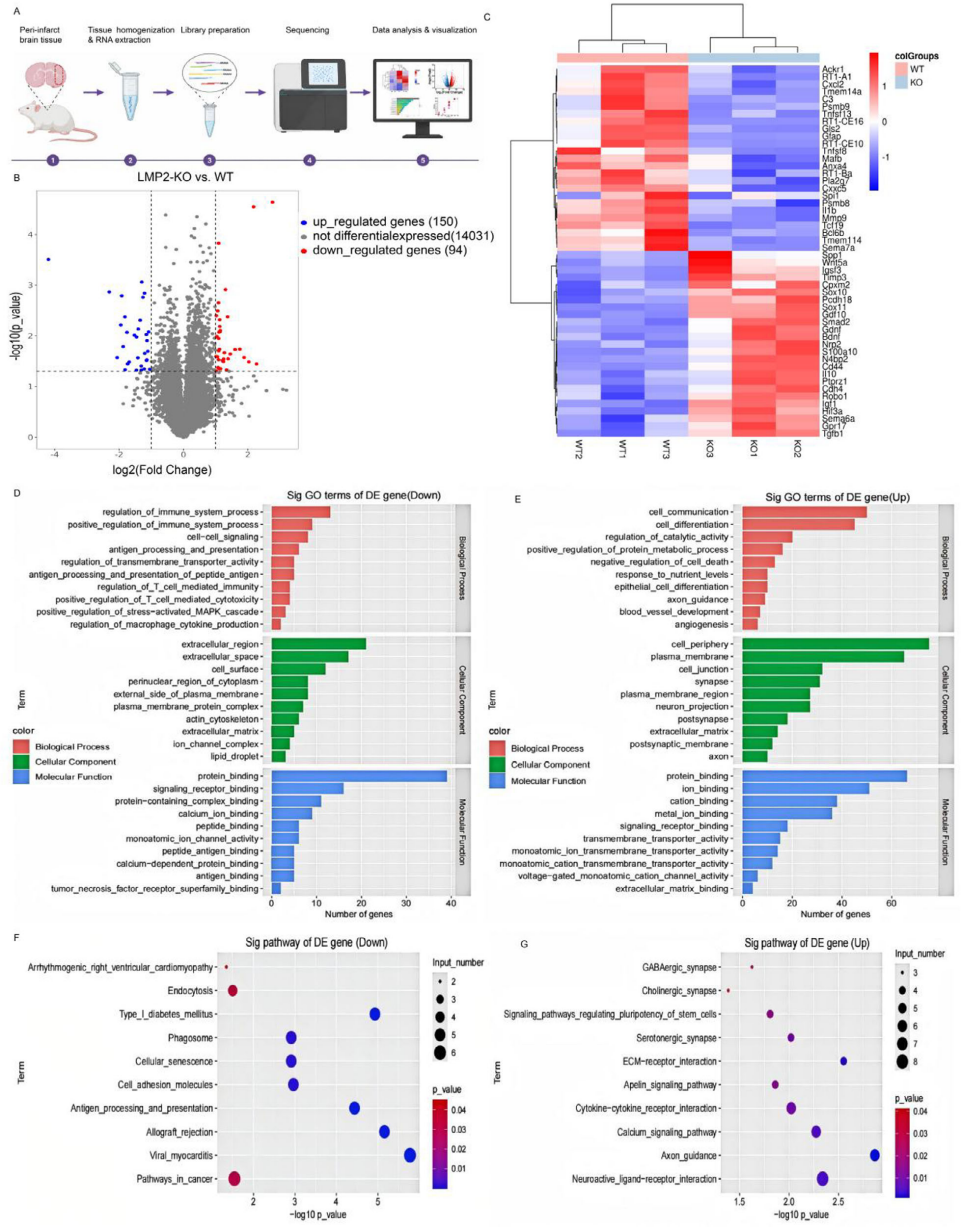
RNA‐seq analysis reveals LMP2‐dependent transcriptional reprogramming following cerebral ischemia. (A) Schematic overview of the RNA‐seq workflow performed on peri‐infarct cortical and striatal tissues collected from WT and LMP2‐KO rats at 3 days after MCAO/R. (B) Volcano plot showing differentially expressed genes (DEGs) between WT and LMP2‐KO rats after MCAO/R. Red dots indicate significantly upregulated genes, blue dots indicate significantly downregulated genes, and gray dots represent genes without significant differential expression. (C) Heatmap showing representative differentially expressed genes (DEGs) between WT and LMP2‐KO rats following MCAO/R. Representative inflammatory genes, including Il1b, Mmp9, and Psmb9, were downregulated in LMP2‐KO rats, whereas genes associated with neuroprotection and tissue repair, including Gdnf, Bdnf, S100a10, Igf1, and Tgfb1, were upregulated. (D‐E) Gene Ontology (GO) enrichment analysis of significantly downregulated (D) and upregulated (E) DEGs in LMP2‐KO rats compared with WT controls. Downregulated genes were predominantly enriched in immune and inflammatory biological processes, whereas upregulated genes were mainly associated with neuronal development, cell communication, and synaptic function. (F,G) Kyoto Encyclopedia of Genes and Genomes (KEGG) pathway enrichment analysis of significantly downregulated (F) and upregulated (G) DEGs. Downregulated pathways were primarily related to immune and inflammatory responses, whereas upregulated pathways were associated with neuronal signaling, axon guidance, extracellular matrix (ECM)‐receptor interaction, calcium signaling, and neuroactive ligand‐receptor interaction. *n* = 3 biological replicates per group.

Previous investigations have established that pharmacological inhibition of LMP2 through stereotactic delivery of LMP2‐shRNA prior to MCAO confers robust neuroprotection against acute ischemic brain injury, indicating that selective targeting of this immunoproteasome subunit represents a promising therapeutic avenue for stroke intervention [17]. To comprehensively elucidate the molecular mechanisms underlying LMP2‐mediated neuroprotective effects, we conducted genome‐wide RNA sequencing (RNA‐seq) coupled with systematic bioinformatic analyses (Figure 3A). Comparative transcriptomic analysis identified 244 differentially expressed genes (DEGs) in peri‐infarct cortical and striatal tissues of LMP2‐KO versus WT rats at 72 h post‐MCAO/reperfusion, comprising 150 upregulated and 94 downregulated transcripts, as illustrated by volcano plot visualization (Figure 3B). Heatmap showing representative differentially expressed genes (DEGs) between WT and LMP2‐KO rats following MCAO/R. Representative inflammatory genes, including *C*3, *Il1b*, *Mmp9*, and *Psmb9*, were downregulated in LMP2‐KO rats, whereas genes associated with neuroprotection and tissue repair, including *Gdnf, Bdnf, S100a10, Igf1*, and *Tgfb1*, were upregulated (Figure 3C).

Gene Ontology (GO) enrichment analysis demonstrated that upregulated genes were predominantly enriched in biological processes governing nervous system development, neurogenesis, axon guidance, and angiogenesis (adjusted *p* < 0.01). Representative transcripts included neurotrophin signaling components (Nrp2, Sox11, Gdnf, Bdnf) and growth modulators (Tgfb1). Cellular component (CC) analysis revealed enrichment in cell periphery, synaptic structures, plasma membrane, and extracellular matrix compartments, exemplified by growth factors (Igf1), matrix regulators (Timp3), and morphogens (Wnt5a). Molecular function (MF) categories encompassed protein binding, metal ion coordination, and receptor signaling activities, with key representatives including synaptic organizers (Agrn), cell adhesion molecules (Cd44), and guidance receptors (Sema6a) (Figure 3D). Conversely, downregulated genes were significantly enriched in biological processes related to immune system regulation, intercellular communication, antigen processing and presentation, and ion homeostasis (adjusted *p* < 0.01). This gene set included immunoproteasome components (Psmb9), complement factors (C3), inflammatory cytokines (Il1b), chemokines (Cxcl2), and TNF superfamily members (Tnfsf8). Cellular component analysis highlighted enrichment in extracellular space, cell surface, and perinuclear cytoplasmic regions, represented by guidance molecules (Sema7a), matrix proteases (Mmp9), annexins (Anxa4), and phospholipases (Pla2g7). Molecular function categories were dominated by protein binding, calcium coordination, and antigen recognition activities, involving MHC class I molecules (RT1‐CE10), TNF ligands (Tnfsf13), Wnt signaling components (Wnt10a), and transcription factors (Mafb) (Figure 3E).

KEGG pathway enrichment analysis corroborated these findings, revealing that LMP2 deficiency significantly suppressed immune‐ and inflammation‐related signaling cascades, including antigen processing and presentation machinery, cell adhesion molecule networks, and cellular senescence pathways, while simultaneously upregulating pathways crucial for neuronal repair and plasticity, such as axon guidance systems, ECM‐receptor interactions, and neuroactive ligand‐receptor signaling (Figure 3F,G). Collectively, these transcriptomic data indicate that LMP2 deletion induces a broad transcriptional reprogramming in the ischemic brain, characterized by coordinated suppression of neuroinflammatory cascades and concurrent activation of neuroprotective and regenerative gene programs, supporting a critical role of LMP2 in coordinating immune responses and neuronal recovery after cerebral I/R injury.

### 3.4 Global LMP2 Knockout Attenuates Pro‐Inflammatory Responses and Preserves Anti‐Inflammatory and Neurotrophic Signaling Following Cerebral Ischemia

**FIGURE 4:**
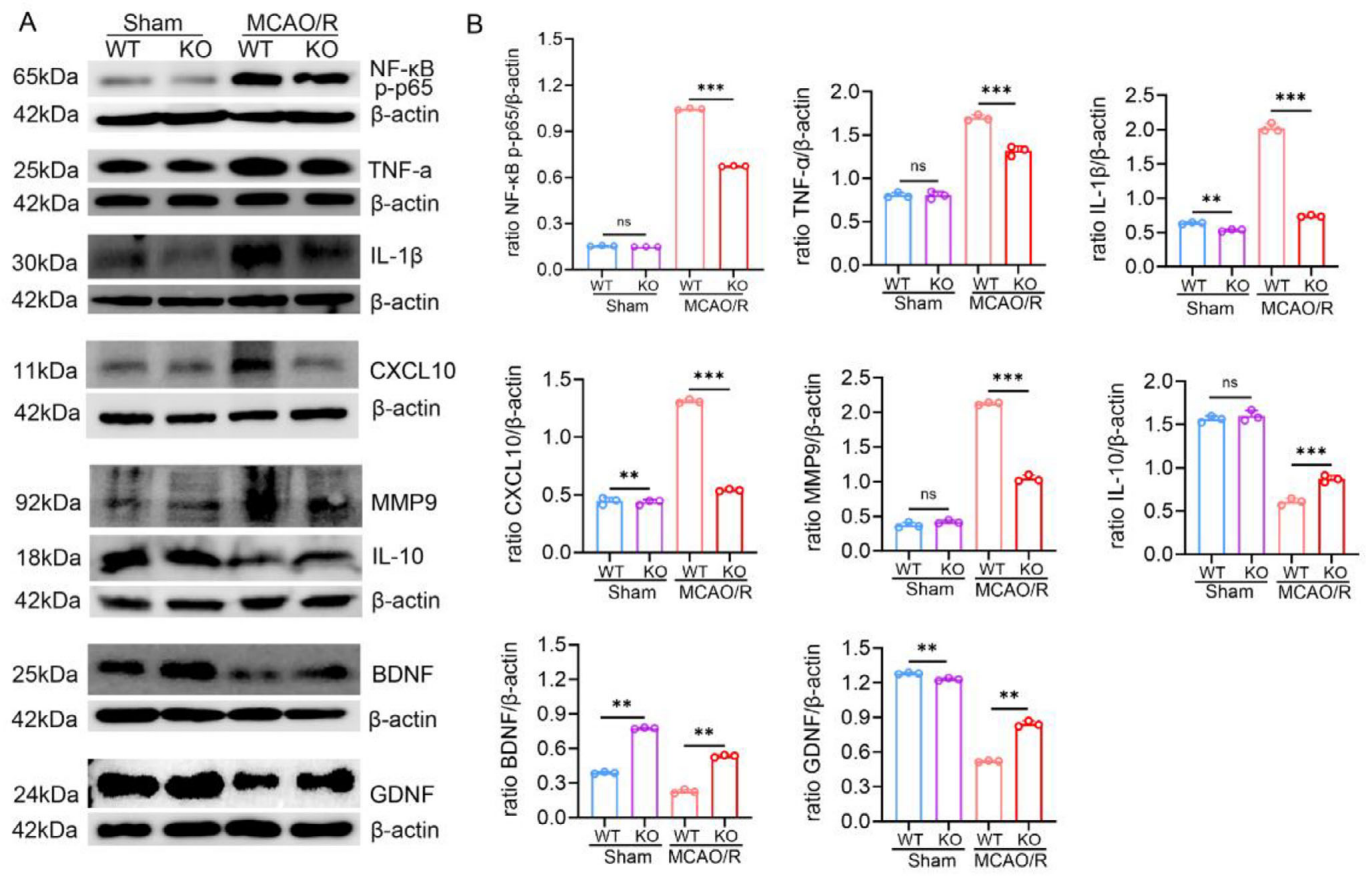
Global LMP2 knockout attenuates pro‐inflammatory responses and preserves anti‐inflammatory and neurotrophic signaling following cerebral ischemia. (A,B) Representative Western blot images and corresponding quantitative analyses of phosphorylated NF‐κB p65 (p‐NF‐κB p65), TNF‐α, IL‐1β, CXCL10, MMP9, IL‐10, BDNF, and GDNF protein expression in WT and LMP2‐KO rats under sham conditions and at 3 days after MCAO/R. Compared with sham‐operated animals, MCAO/R increased the expression of p‐NF‐κB p65, TNF‐α, IL‐1β, CXCL10, and MMP9, while reducing IL‐10, BDNF, and GDNF protein levels. Compared with WT rats, LMP2‐KO rats exhibited lower expression of p‐NF‐κB p65, TNF‐α, IL‐1β, CXCL10, and MMP9, together with higher expression of IL‐10, BDNF, and GDNF following MCAO/R. *n* = 3 rats per group. Data are presented as mean ± SD from three independent experiments using one‐way ANOVA with LSD's post hoc test. ns, not significant; ^**^ *p* < 0.01, ^***^ *p* < 0.001.

Reactive astrogliosis represents a hallmark pathological feature of cerebral ischemia, characterized by amplified inflammatory signaling cascades and excessive production of pro‐inflammatory cytokines, including IL‐1β and TNF‐α [6, 33]. These inflammatory mediators contribute substantially to secondary neuronal injury and represent critical therapeutic targets for CNS disorders [34]. To validate our RNA‐seq findings at the protein level, we performed comprehensive Western blot analysis of key inflammatory and neurotrophic markers. Consistent with our transcriptomic data demonstrating downregulation of pro‐inflammatory genes (NF‐κB, TNF‐α, IL‐1β, CXCL10, and MMP9) and upregulation of neurotrophic factors (GDNF and BDNF) in LMP2‐KO rats (Figure 3C), proteomic analysis confirmed these transcriptional alterations. At 72 h post‐MCAO/reperfusion, cerebral ischemia induced a robust inflammatory response in wild‐type animals, as evidenced by significant elevation of phosphorylated NF‐κB p65, TNF‐α, IL‐1β, CXCL10, and MMP9 protein levels compared to sham‐operated controls. Notably, this ischemia‐induced pro‐inflammatory protein upregulation was markedly attenuated in LMP2‐KO animals, demonstrating the critical role of LMP2 in orchestrating post‐ischemic neuroinflammation (Figure 4A).

In stark contrast to the suppression of inflammatory mediators, LMP2 deficiency significantly enhanced the expression of neuroprotective and neurotrophic factors. Quantitative analysis revealed substantially increased BDNF and GDNF protein levels in LMP2‐KO + MCAO/R animals compared to their wild‐type counterparts (*p* < 0.01; Figure 4A,B). This reciprocal regulation pattern, simultaneous suppression of inflammatory cascades and enhancement of neurotrophic signaling, suggests that LMP2 deletion promotes a favorable microenvironmental shift from neurodestructive to neuroprotective conditions.

Collectively, these complementary transcriptomic and proteomic findings provide compelling evidence that genetic ablation of LMP2 fundamentally reprograms the post‐ischemic brain response by suppressing deleterious pro‐inflammatory cytokine production while concurrently promoting beneficial neurotrophic and anti‐inflammatory factor expression. This dual regulatory mechanism underlies the neuroprotective efficacy of LMP2 targeting and supports its therapeutic potential for cerebral ischemia‐reperfusion injury.

### 3.5 Astrocyte‐Specific LMP2 Deletion Protects Against Cerebral Ischemia/Reperfusion Injury in Mice

**FIGURE 5:**
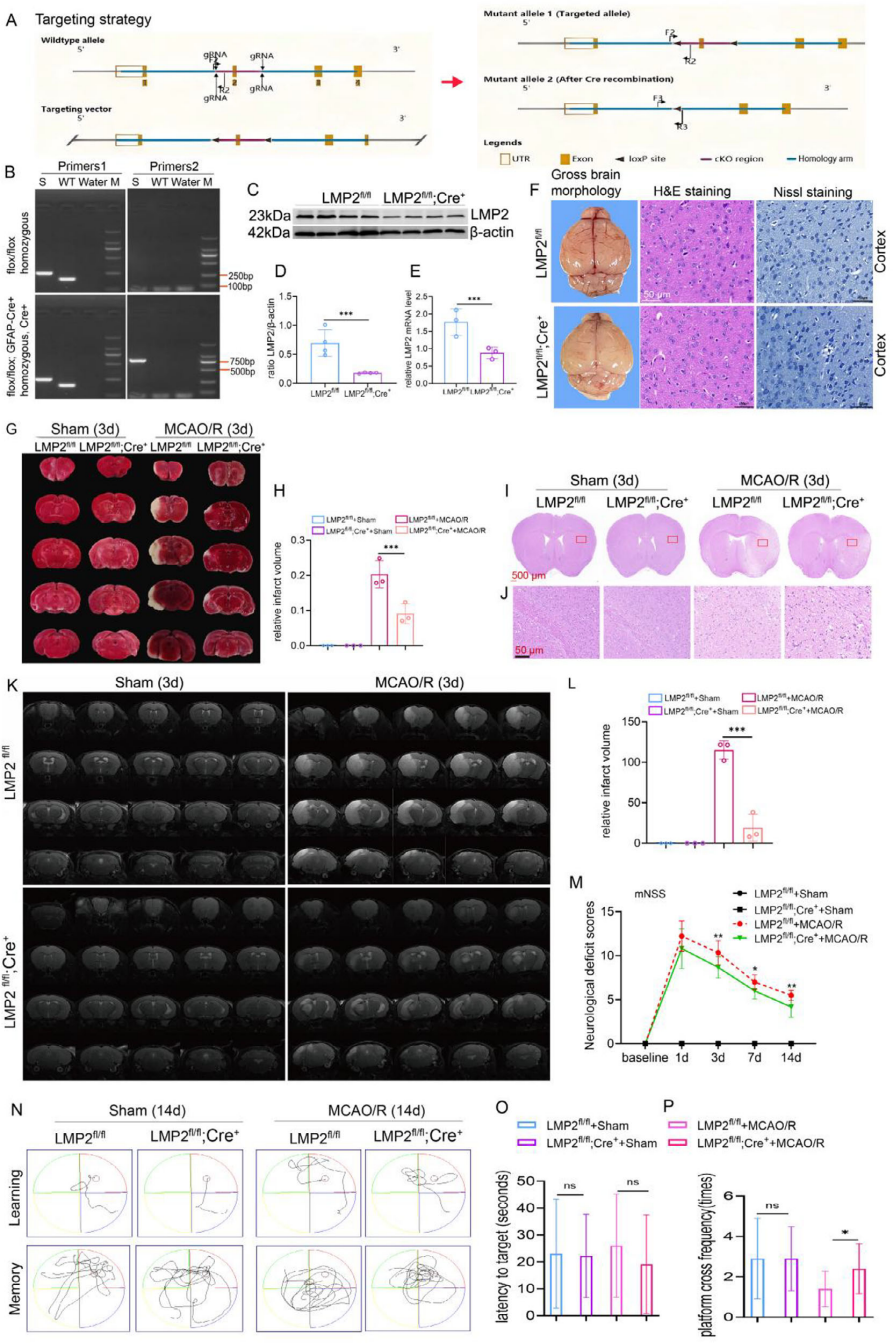
Astrocyte‐specific LMP2 deletion protects against cerebral ischemia/reperfusion injury in mice. (A) Schematic illustration of the strategy used to generate astrocyte‐specific LMP2 conditional knockout (LMP2^fl/fl^;Cre^+^) mice. LoxP sites were inserted flanking the target region, and Cre recombination excised the floxed allele in astrocytes. (B) PCR‐based genotyping of LMP2^fl/fl^ and LMP2^fl/fl^;Cre^+^ mice using primer set 1 and primer set 2. (C–E) Validation of astrocyte‐specific LMP2 deletion. (C,D) Representative Western blot images and corresponding quantification showing reduced LMP2 protein expression in the cerebral cortex of LMP2^fl/fl^;Cre^+^ mice compared with LMP2^fl/fl^ littermate controls. Protein levels were normalized to β‐actin. n = 4 mice per group. Data are presented as mean ± SD from three independent experiments using unpaired two‐tailed Student's t test. (E) Relative mRNA expression levels of LMP2. *n* = 3 mice per group. Data are presented as mean ± SD from three independent experiments using unpaired two‐tailed Student's t test. (F) Representative images of gross brain morphology, hematoxylin and eosin (H&E), and Nissl staining showing no obvious structural or histological abnormalities in the cortex between LMP2^fl/fl^ and LMP2^fl/fl^;Cre^+^ mice under physiological conditions. *n* = 3 mice per group. (G–H) Representative TTC‐stained coronal sections and corresponding quantification of infarct volumes at 3 days after MCAO/R. *n* = 3 mice per group. Data are presented as mean ± SD from three independent experiments using one‐way ANOVA with LSD's post hoc test. ^***^ *p* < 0.001. (I‐J) Representative H&E staining images at 3 days post‐MCAO/R, showing reduced neuronal damage and tissue loss in LMP2^fl/fl^;Cre^+^ mice compared with LMP2^fl/fl^ mice. Panel J shows an enlarged view of the region indicated by the red box in panel I. *n* = 3 mice per group. (K‐L) Representative T2‐weighted MRI images and corresponding quantification of infarct volumes. *n* = 3 mice per group. Data are presented as mean ± SD from three independent experiments using one‐way ANOVA with LSD's post hoc test. ^***^ *p* < 0.001. (M) Longitudinal neurological assessment using mNSS. *n* = 10 mice per group. (N–P) Morris water maze test evaluating spatial learning and memory at 14 days post‐MCAO/R. Representative swimming traces (N), escape latency (O), and platform crossing frequency (P) indicate improved cognitive performance in LMP2^fl/fl^;Cre^+^ mice. *n* = 10 mice per group. Scale bars = 500 µm (I, upper panels), 50 µm (J,F). Data are presented as mean ± SD, repeated measures ANOVA with LSD's post hoc testing. ns, not significant; ^*^ *p* < 0.05, ^**^ *p* < 0.01, ^***^ *p* < 0.001.

Reactive astrocytes activate innate immune pathways and aggravate secondary ischemic injury [35]. Given their crucial role across multiple spatial and temporal stages of stroke pathology, targeting reactive astrocytes represents a promising therapeutic strategy [5]. Building on our findings that complete LMP2 knockout reduces infarct volume and attenuates astrocytic activation, we investigated the specific role of astrocytic LMP2 in ischemic brain injury. To this end, LMP2^flox/flox^ mice were crossed with GFAP‐Cre transgenic mice to generate astrocyte‐specific LMP2 conditional knockout mice (LMP2^flox/flox^; GFAP‐Cre^+^, abbreviation LMP2^fl/fl^; Cre^+^), with LMP2^flox/flox^ littermates lacking Cre recombinase (LMP2^flox/flox^; GFAP‐Cre^−,^ abbreviation LMP2^fl/fl^) serving as controls (Figure 5A,B). Western blot analysis revealed a robust reduction in LMP2 protein expression in LMP2^fl/fl^;Cre^+^ mice compared with LMP2^fl/fl^ controls (mean intensity: 0.17 ± 0.01 vs. 0.69 ± 0.20, *p* < 0.001) (Figure 5C,D). Consistently, RT‐ qPCR analysis confirmed a marked decrease in LMP2 mRNA expression in LMP2^fl/fl^;Cre^+^ mice (2^−△△CT^: 0.88 ± 0.29 vs. 1.77 ± 0.40, *p* < 0.001) (Figure 5E). The relatively moderate reduction in total LMP2 protein levels likely reflects cellular heterogeneity in whole‐brain lysates, as astrocytes constitute only a subset of total brain cells.

To exclude potential confounding effects of GFAP‐Cre expression under physiological conditions, we evaluated baseline proteasome composition, neural cell integrity, and inflammatory status. Expression levels of representative immunoproteasome subunits (LMP7 and MECL1) and the constitutive proteasome subunit PSMB6 were comparable between LMP2^fl/fl^ and LMP2^fl/fl^;Cre^+^ mice (Figure S1A,B). Consistently, RT‐qPCR analysis revealed no significant differences in mRNA expression levels of Psmb8 (LMP7), Psmb10 (MECL‐1), and Psmb6 between groups (Figure S1C), indicating that astrocyte‐specific LMP2 deletion does not trigger compensatory alterations in other proteasome subunits under basal conditions.

Immunofluorescence staining showed no apparent differences in NeuN^+^ neurons, IBA1^+^ microglia, or GFAP^+^ astrocytes (Figure S1D,E), and the protein levels of pro‐inflammatory cytokines (IL‐1β and TNF‐α) were similarly expressed between groups (Figure S1F,G). Importantly, these findings indicate that GFAP‐Cre expression does not cause overt baseline neurotoxicity or off‐target inflammatory effects in this model. In addition, both LMP2^fl/fl^ and LMP2^fl/fl^; Cre^+^ mice developed normally without signs of neurological dysfunction, infertility, or behavioral abnormalities. H&E and Nissl staining revealed comparable cortex cytoarchitecture between genotypes (Figure 5F), and no gross morphological abnormalities were detected in major organs (heart, liver, lung, kidney, and spleen), indicating that astrocyte‐specific LMP2 deletion did not cause systemic developmental defects (Figure S2).

Having established the astrocyte‐specific LMP2 conditional knockout model, we next examined the cellular distribution and temporal dynamics of LMP2 expression following cerebral ischemia. Under physiological conditions, LMP2 expression was relatively low throughout the brain. Given the limited basal GFAP expression in the cortex, we selected the corpus callosum to optimize astrocyte visualization. Immunofluorescence analysis revealed weak but detectable LMP2 signals with partial co‐localization in GFAP^+^ astrocytes, which was markedly reduced in LMP2^fl/fl^;Cre^+^ mice, confirming the efficiency of astrocyte‐specific deletion (Figure S3A,B). Following MCAO/R, LMP2 expression was markedly upregulated in the peri‐infarct cortex and striatum at 3 days post‐reperfusion, with prominent co‐localization with GFAP^+^ reactive astrocytes. Quantitative analysis demonstrated significant increases in both the number of LMP2^+^/GFAP^+^ cells and the proportion of GFAP^+^ astrocytes expressing LMP2, which was significantly attenuated in LMP2^fl/fl^;Cre^+^ mice (Figure S3C,D). To further characterize the cell‐type distribution of LMP2 after ischemia, co‐immunostaining with multiple cellular markers was performed. LMP2 expression was predominantly associated with GFAP^+^ astrocytes (∼69.6%), with lower representation in CD31^+^ endothelial cells (∼17.3%), IBA1^+^ microglia (∼9.3%), and NeuN^+^ neurons (∼11.6%) (Figure S3E,F). Notably, these proportions reflect the relative distribution of LMP2^+^ cells across different cell populations rather than mutually exclusive categories. Western blot analysis corroborated the immunofluorescence findings, revealing low LMP2 protein expression under sham conditions that increased markedly following MCAO/R, peaked at 3 days post‐reperfusion, and subsequently declined at later time points (7 and 14 days) (Figure S3G,H). Moreover, LMP2 expression was significantly reduced in LMP2^fl/fl^;Cre^+^ mice compared with LMP2^fl/fl^ controls at both 1 and 3 days post‐MCAO/R, providing additional confirmation of efficient astrocyte‐specific deletion in ischemia‐affected regions (Figure S3I,J).

To further characterize the temporal dynamics of astrocyte activation following ischemia, we examined GFAP expression, a marker of astrocyte reactivity, across multiple timepoints after MCAO/R. Western blot analysis revealed that GFAP levels were markedly increased in the peri‐infarct cortex and striatum of the MCAO/R group compared to sham controls (Figure S3K,L). Interestingly, the temporal profile of GFAP expression closely paralleled that of LMP2 (Figure S3G,H), with levels peaking at 3 days post‐reperfusion and gradually declining at later timepoints (7 and 14 days) (Figure S3M,N). Notably, astrocyte‐specific deletion of LMP2 significantly attenuated ischemia‐induced GFAP upregulation across multiple timepoints, indicating reduced astrocyte reactivity in the absence of LMP2. Importantly, the temporal profile of GFAP expression closely paralleled that of LMP2, supporting the selection of the 3‐day timepoint as a representative stage of peak astrocyte activation for subsequent mechanistic and transcriptomic analyses. Taken together, these results demonstrate that LMP2 is robustly induced following cerebral ischemia/reperfusion, with preferential enrichment in astrocytes, supporting its association with reactive astrocytes during the acute phase of injury.

Functionally, TTC staining revealed a significant reduction in infarct volume in LMP2^fl/fl^; Cre^+^ mice compared to LMP2^fl/fl^ controls at 3 days post‐MCAO/R (infarct volume: 9.1 ± 0.3% vs. 20.3 ± 0.4%, *p* < 0.001) (Figure 5G,H). Consistent with this, H&E staining at the same timepoint showed markedly reduced neuronal damage and tissue loss in the cortex and striatum of LMP2^fl/fl^;Cre^+^ mice compared to LMP2^fl/fl^ mice (Figure 5I,J). Concordantly, T2‐weighted MRI analysis showed no ischemic lesions in sham‐operated mice, whereas clear infarction was observed in MCAO/R groups. Notably, LMP2^fl/fl^; Cre^+^ mice exhibited substantially smaller infarct volumes than LMP2^fl/fl^ controls (19.1 ± 16.6 mm^3^ vs. 115.0 ± 11.2 mm^3^, *p* <0.001) (Figure 5K,L).

Neurological function assessed by the modified Neurological Severity Score (mNSS) revealed that sham animals exhibited no detectable deficits, whereas MCAO/R induced marked impairments in motor, sensory, and balance functions. Importantly, LMP2^fl/fl^; Cre^+^ mice showed significantly improved neurological outcomes compared to LMP2^fl/fl^ control at 3, 7 and 14 days post‐reperfusion (mNSS 3d: 8.7 ± 1.2 vs.10.3 ± 1.4, *p* = 0.008; 7d: 6.0 ± 0.9 vs. 7.0 ± 0.8, *p* = 0.022; 14d: 4.2 ± 1.2 vs. 5.5 ± 0.6, *p* = 0.009) (Figure 5 M). Cognitive performance was evaluated using the Morris water maze at 14 days post‐stroke. While MCAO/R mice exhibited impaired spatial learning and memory, LMP2^fl/fl^; Cre^+^ mice subjected to MCAO/R crossed the target platform significantly more frequently than LMP2^fl/fl^ MCAO/R mice, despite comparable escape latencies (Figure 5N–P).

Collectively, these findings indicate that astrocyte‐specific deletion of LMP2 attenuates ischemic brain injury and promotes both neurological and cognitive recovery following cerebral I/R, highlighting a critical role for astrocytic LMP2 in post‐ischemic pathophysiology.

### 3.6 Astrocyte‐Specific LMP2 Deletion Modulates Astrocyte Activation and Functional States Following Cerebral Ischemia in Mice

**FIGURE 6:**
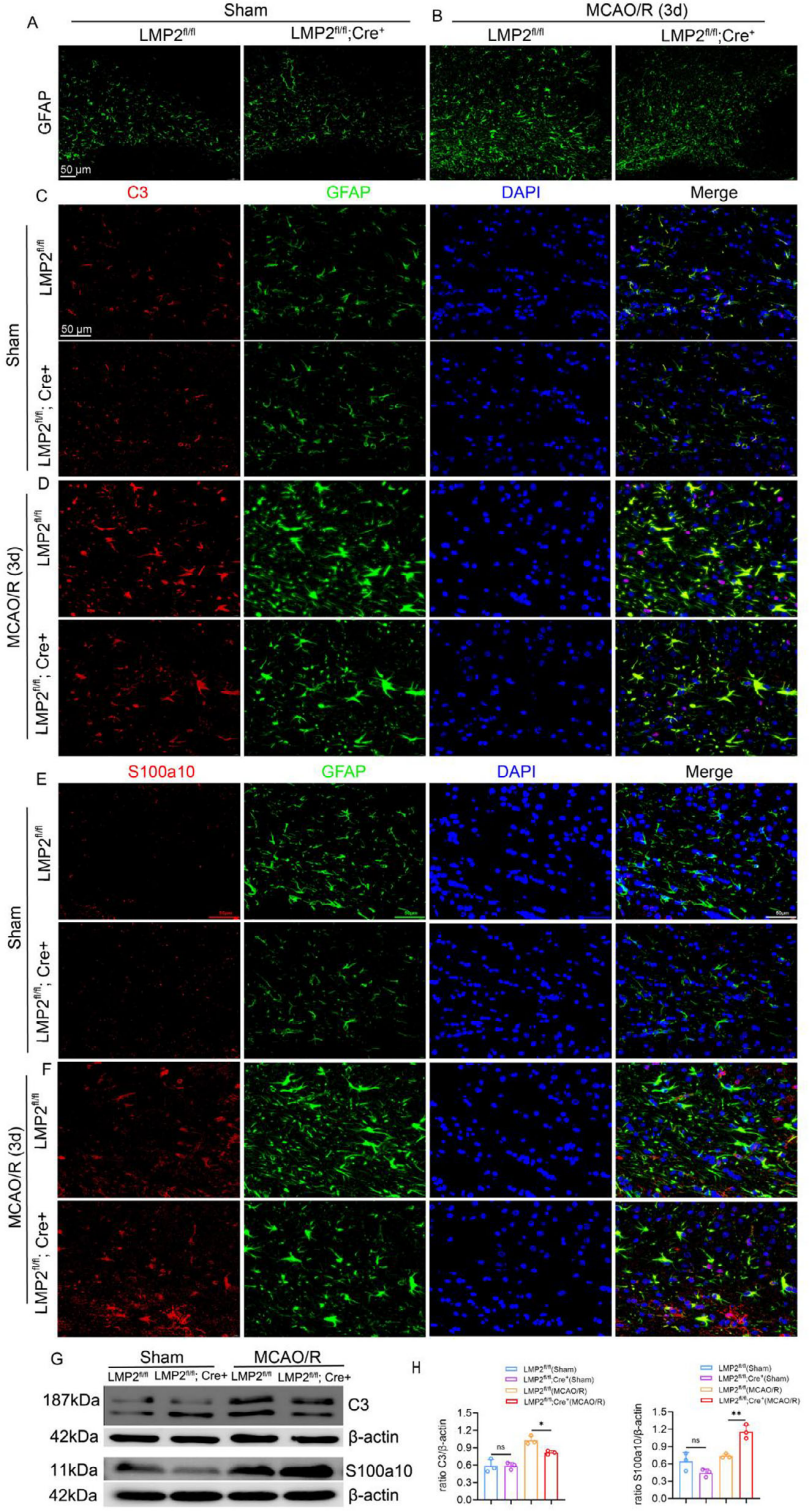
Astrocyte‐specific LMP2 deletion modulates astrocyte activation and functional states following cerebral ischemia in mice. (A,B) Representative immunofluorescence staining of GFAP (green) showing astrocyte activation in the cortex and striatum of LMP2^fl/f^l and LMP2^fl/fl^;Cre^+^ mice under sham and MCAO/R conditions (3 days post‐reperfusion). Scale bars = 50 µm. *n* = 3 mice per group. (C–F) Representative immunofluorescence images showing C3 and S100a10 expression in GFAP^+^ astrocytes in the peri‐infarct cortex. C3 and S100a10 signals are shown in red, GFAP in green, and nuclei are counterstained with DAPI (blue). Co‐localization analysis indicates that both markers are expressed in reactive astrocytes under ischemic conditions. *n* = 3 mice per group. (G,H) Representative Western blot images and quantitative analysis of C3 and S100a10 protein expression in the sham and MCAO/R groups. *n* = 3 mice per group. Data are presented as mean ± SD from three independent experiments using one‐way ANOVA with LSD's post hoc test. ns: not significant; ^*^ *p* < 0.05, ^**^ *p* < 0.01.

Astrocytes are key regulators of neuroinflammation and neuronal survival after ischemic injury and exhibit diverse, context‐dependent functional states in response to pathological stimuli [8, 36]. To determine whether LMP2 influences astrocyte reactivity, we examined GFAP expression and functional state–associated markers in LMP2^fl/fl^ and LMP2^fl/fl^; Cre^+^ mice following MCAO/R. Under sham conditions, GFAP immunofluorescence intensity was comparable between genotypes, indicating baseline quiescence (Figure 6A). After ischemia/reperfusion, GFAP^+^ astrocytes were markedly increased in peri‐infarct regions of LMP2^fl/fl^ MCAO/R mice, whereas this activation was significantly attenuated in LMP2^fl/fl^; Cre^+^ MCAO/R mice (Figure 6B). Interestingly, microglial activation showed a similar trend, as IBA1 immunoreactivity and morphological transformation were reduced in LMP2^fl/fl^; Cre^+^ MCAO/R mice compared with controls (Figure S4), suggesting that astrocytic LMP2 may indirectly modulate microglial reactivity.

To further characterize astrocyte functional responses, we assessed the expression of C3 and S100a10, which are commonly associated with pro‐inflammatory and reparative astrocyte programs, respectively. Immunohistochemical (IHC) staining revealed that C3 immunoreactivity was markedly reduced in LMP2^fl/fl^; Cre^+^ MCAO/R mice compared with LMP2^fl/fl^ MCAO/R controls, whereas S100a10 expression showed the opposite trend, with notable enhancement (Figure S5). Consistent results were obtained by immunofluorescence staining (Figure 6C–F), where C3 expression was strongly induced in the LMP2^fl/fl^ MCAO/R group but markedly reduced in LMP2^fl/fl^; Cre^+^ MCAO/R mice. In contrast, S100a10 expression was significantly increased in the conditional knockout mice. Co‐localization analysis confirmed that both C3 and S100a10 were predominantly expressed in GFAP^+^ astrocytes. Furthermore, Western blot analysis corroborated these findings: both C3 and S100a10 protein levels were elevated after MCAO/R compared with sham controls, whereas LMP2^fl/fl^; Cre^+^ MCAO/R mice exhibited significantly decreased C3 and increased S100a10 expression relative to LMP2^fl/fl^ MCAO/R counterparts (Figure 6G,H).

Overall, these results demonstrate that astrocyte‐specific deletion of LMP2 reshapes astrocyte functional states following cerebral ischemia, suppressing pro‐inflammatory astrocyte–associated responses while enhancing reparative and neuroprotective programs. These findings suggest that LMP2 serves as a key regulator of astrocytic reactivity and glial crosstalk in post‐ischemic neuroinflammation.

### 3.7 Single‐Cell Transcriptomic Analysis Reveals Heterogeneous and Continuum‐Like Astrocyte States Following Cerebral Ischemia

To further address the limitation of the traditional binary classification of astrocyte activation and to better characterize astrocyte heterogeneity, we performed single‐cell RNA sequencing of peri‐infarct cortex and striatum 3 days after cerebral ischemia/reperfusion. Unsupervised clustering identified multiple major brain cell populations, including astrocytes, microglia, oligodendrocytes, endothelial cells, and other cell types, reflecting the cellular complexity of the ischemic microenvironment (Figure S6A,B).

Focusing on astrocytes, subclustering analysis identified six transcriptionally distinct astrocyte subpopulations (C1‐C6), each defined by characteristic gene expression profiles and associated with diverse functional programs, including metabolic regulation, neuroprotective/metabolic responses, inflammatory activation, stress adaptation, neurorepair, and antigen presentation (Figure S6C). Comparative analysis between LMP2^fl/fl^;Cre^+^ MCAO/R and LMP2^fl/fl^ MCAO/R groups revealed that astrocyte‐specific LMP2 deletion reshaped astrocyte composition, characterized by a reduction in inflammation‐associated clusters and a relative increase in clusters enriched for neuroprotective and metabolic gene signatures (Figure S6C,D).

Importantly, canonical markers previously associated with pro‐inflammatory and reparative astrocyte features (e.g., C3 and S100a10) were broadly distributed across multiple astrocyte subclusters rather than being confined to discrete populations (Figure S6E). Both markers exhibited graded and overlapping expression patterns across clusters associated with inflammatory, metabolic, and reparative programs, indicating that they represent partial functional features rather than mutually exclusive astrocyte states.

Taken together, these findings support a model in which astrocytes exist along a dynamic continuum of transcriptional states. Within this framework, LMP2 does not drive a binary phenotypic switch but instead modulates the distribution of astrocyte states along this continuum, representing a coordinated shift in inflammatory and reparative transcriptional programs following ischemic injury.

### 3.8 Astrocyte‐Specific LMP2 Conditional Knockout Modulates Inflammatory and Neurotrophic Responses Following Cerebral Ischemia in Mice

**FIGURE 7:**
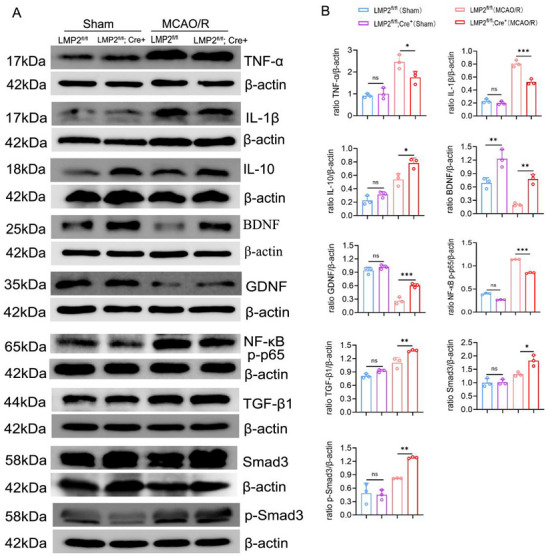
Astrocyte‐specific LMP2 deletion modulates inflammatory and neurotrophic responses and associated signaling pathways following cerebral ischemia in mice. (A) Representative Western blot images showing protein expression levels of TNF‐α, IL‐1β, IL‐10, BDNF, GDNF, phosphorylated NF‐κB p65 (NF‐κB p‐p65), TGF‐β1, Smad3, and phosphorylated Smad3 (p‐Smad3) in the peri‐infarct cortex and striatum tissues from LMP2^fl/fl^ and LMP2^fl/fl^; Cre^+^ mice under sham and MCAO/R (3 days post‐reperfusion) conditions. (B) Quantitative analysis of protein expression levels normalized to β‐actin in the indicated groups. *n* = 3 mice per group. Data are presented as mean ± SD from three independent experiments using one‐way ANOVA with LSD's post hoc test. ns, not significant; ^*^ *p* < 0.05, ^**^ *p* < 0.01, ^***^ *p* < 0.001.

Given that astrocytic LMP2 contributes to glial activation following ischemic stroke, we next investigated whether astrocytic LMP2 regulates the production of inflammatory mediators in vivo. Western blot analysis showed that, compared with sham controls, MCAO/R markedly increased the levels of pro‐inflammatory cytokines TNF‐α and IL‐1β, while significantly reducing the expression of anti‐inflammatory and neurotrophic factors, including IL‐10, BDNF, and GDNF (*p* < 0.05). Notably, astrocyte‐specific deletion of LMP2 significantly attenuated the ischemia‐induced upregulation of TNF‐α and IL‐1β, while restoring the levels of IL‐10, BDNF, and GDNF compared with LMP2^fl/fl^ MCAO/R mice (Figure 7A,B).

Previous studies have identified both the NF‐κB and TGF‐β/Smad signaling pathways as key regulators of neuroinflammatory responses under pathological conditions [8]. Consistent with our previous findings that LMP2 suppression reduces NF‐κB p65 activation following focal cerebral ischemia in rats [17]. We next examined whether astrocytic LMP2 modulates these signaling pathways in vivo. Western blot analysis revealed that MCAO/R significantly increased the levels of phosphorylated NF‐κB p65 (p‐p65), TGF‐β1, Smad3, and phosphorylated Smad3 (p‐Smad3) compared with sham controls. Importantly, astrocyte‐specific LMP2 deletion markedly reduced NF‐κB p65 phosphorylation, while further enhancing the expression of TGF‐β1, Smad3, and p‐Smad3 (*p* < 0.05; Figure 7A,B). These findings were further corroborated by immunofluorescence analysis. MCAO/R induced robust activation of NF‐κB signaling, as evidenced by increased nuclear accumulation of NF‐κB p65, whereas this nuclear translocation was markedly attenuated in LMP2^fl/fl^;Cre^+^ mice (Figure S7A–C). In contrast, Smad3 signaling exhibited an opposite pattern. MCAO/R enhanced the nuclear accumulation of p‐Smad3, and this effect was further increased in LMP2^fl/fl^;Cre^+^ mice (Figure S7D–F), indicating enhanced activation of the TGF‐β1/Smad3 pathway.

Together, these findings suggest that astrocytic LMP2 is associated with the regulation of inflammatory signaling following cerebral ischemia, promoting NF‐κB‐dependent pro‐inflammatory activation while constraining TGF‐β1/Smad3‐associated reparative signaling. Consistent with this, its conditional deletion shifts the balance of these pathways toward a more anti‐inflammatory and neuroprotective milieu after cerebral ischemia/reperfusion injury.

### 3.9 In Vitro Effects of LMP2 Inhibition on Astrocyte Reactive States, Functional Responses, and Cell Fate Under Ischemic‐Like Conditions

Our previous study demonstrated that oxygen–glucose deprivation/reperfusion (OGD/R) induces LMP2 upregulation and enhances proteasome activity in astrocytes under ischemic‐like conditions [27]. In the present study, we utilized the CTX‐TNA2 rat astrocytic cell line as an in vitro model to further characterize astrocyte responses to ischemic stress. Consistent with in vivo observations, OGD/R exposure induced marked astrocyte activation, accompanied by broad inflammatory and reparative alterations, together with impaired cellular viability. Using CCK‐8 assays, we identified 2 h of oxygen–glucose deprivation as the optimal duration for subsequent OGD/R experiments (Figure S8A,B).

Immunofluorescence analysis revealed that both C3 and S100a10 immunoreactivity were relatively low under basal conditions but were increased following OGD/R treatment, suggesting induction of heterogeneous astrocyte reactive programs under ischemic‐like stress (Figure S8C–F). Consistently, Western blot analysis confirmed significant upregulation of both C3 and S100a10 protein levels in OGD/R‐treated CTX‐TNA2 astrocytes compared with controls (*p* < 0.001; Figure S8G,H), indicating broad astrocyte reactive state remodeling under ischemic‐like conditions. In parallel, OGD/R markedly enhanced inflammatory signaling programs, including TNF‐α, IL‐1β, and CXCL10, while reducing the levels of anti‐inflammatory and neurotrophic factors such as IL‐10, BDNF, and GDNF (Figure S8I,J). Furthermore, OGD/R impaired CTX‐TNA2 astrocyte viability and promoted apoptosis, as evidenced by increased expression of Bax and cleaved caspase‐3 (p17), along with a decreased Bcl2/Bax ratio (Figure S8K–L). In summary, these results demonstrate that OGD/R induces extensive astrocyte functional reprogramming characterized by enhanced inflammatory responses, impaired reparative programs, and increased apoptotic susceptibility under ischemic‐like conditions.

**FIGURE 8:**
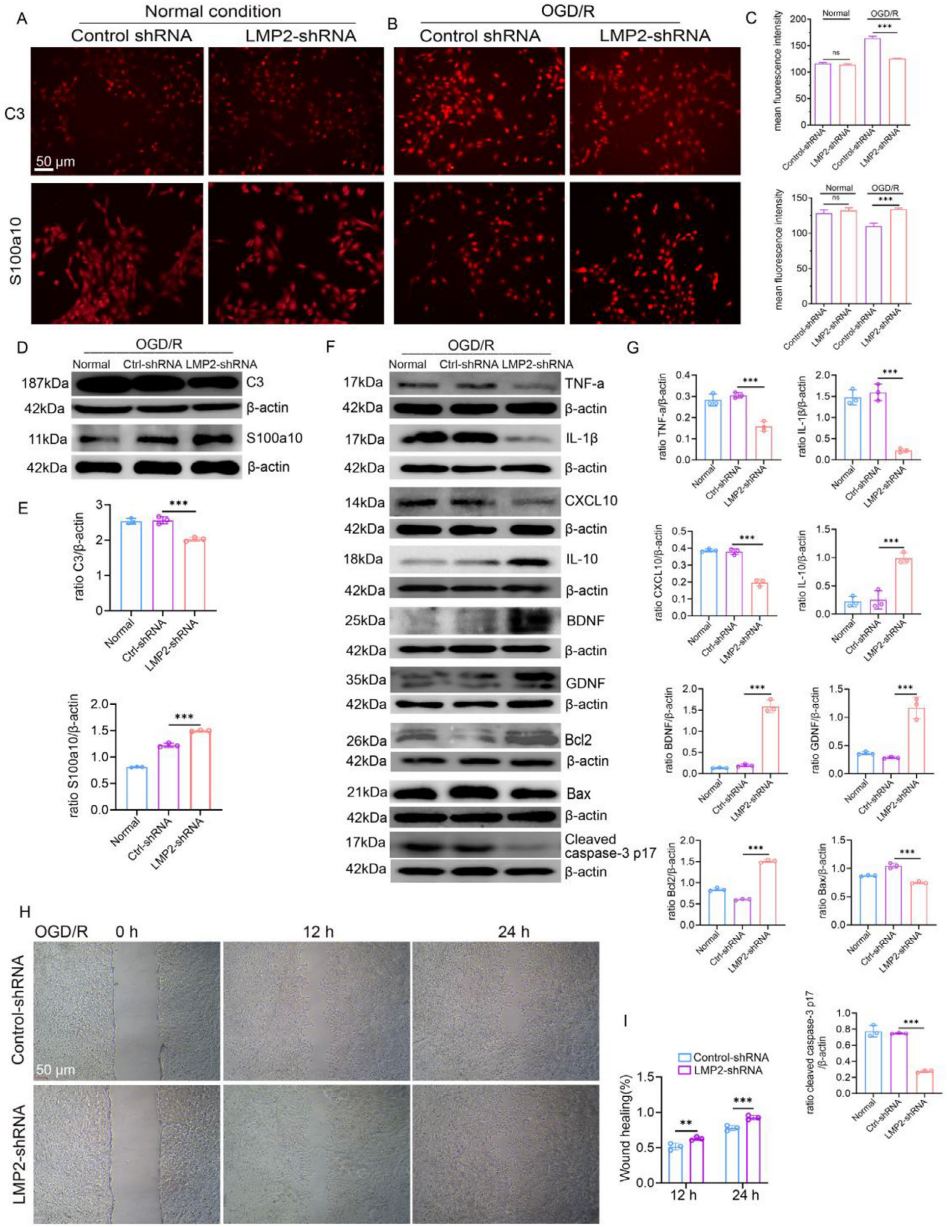
LMP2 inhibition modulates CTX‐TNA2 astrocyte phenotype, inflammatory profile, and functional properties in vitro. (A–C) Representative immunofluorescence images and corresponding quantification of C3 and S100a10 expression in CTX‐TNA2 astrocytes transfected with control shRNA or LMP2 shRNA under normal conditions and following OGD/R. Scale bar = 50 µm. (D,E) Representative Western blot images and corresponding quantification of C3 and S100A10 protein expression in non‐transfected CTX‐TNA2 astrocytes (Normal) and in control shRNA‐ or LMP2 shRNA‐transfected astrocytes following OGD/R. (F,G) Representative Western blot images and corresponding quantitative analyses of TNF‐α, IL‐1β, CXCL10, IL‐10, BDNF, GDNF, Bcl‐2, Bax, and cleaved caspase‐3 p17 protein expression in non‐transfected CTX‐TNA2 astrocytes (Normal) and in control shRNA‐ or LMP2 shRNA‐transfected astrocytes following OGD/R. (H,I) Representative images and quantitative analysis of wound healing assays showing astrocyte migration in CTX‐TNA2 cells under OGD/R conditions at the indicated time points (0, 12, and 24 h). LMP2 knockdown significantly enhanced wound closure rates, indicating increased migratory capacity compared with control‐shRNA cells. Scale bar = 50 µm. Normal: non‐transfected CTX‐TNA2 astrocytes. OGD/R: oxygen‐glucose deprivation/reoxygenation. Data are presented as mean ± SD from three independent experiments using one‐way ANOVA with LSD's post hoc test. ^**^ *p* < 0.01, ^***^ *p* < 0.001.

To further elucidate the regulatory role of LMP2 in astrocyte activation and cell fate, we established an in vitro OGD/R model and performed lentiviral‐mediated LMP2 knockdown. Efficient silencing was confirmed at both mRNA and protein levels (Figure S9A–F). Under OGD/R conditions, control‐shRNA CTX‐TNA2 cells exhibited marked induction of C3, whereas LMP2 silencing significantly attenuated C3 expression and concomitantly increased S100a10 levels, as demonstrated by both immunofluorescence and western blot analyses (Figure 8A–E). These findings indicate that LMP2 regulates inflammatory–reparative astrocyte state transitions. Consistent with this shift in astrocyte functional states, LMP2 knockdown markedly suppressed pro‐inflammatory mediators (TNF‐α, IL‐1β, and CXCL10) while enhancing anti‐inflammatory and neurotrophic factors (IL‐10, BDNF, and GDNF) under OGD/R conditions (Figure 8F,G), consistent with a coordinated shift from inflammatory toward reparative astrocyte programs. In addition, LMP2 silencing significantly inhibited apoptosis, as evidenced by increased Bcl‐2 expression and reduced Bax and cleaved caspase‐3 levels (Figure 8F,G). Functionally, scratch wound assays revealed enhanced migratory capacity in LMP2‐shRNA–treated astrocytes, with significantly higher wound closure rates at both 12 and 24 h compared with control cells (Figure 8H,I). Taken together, these findings demonstrate that LMP2 inhibition shifts astrocyte reactive states toward reparative programs while suppressing inflammatory responses, thereby promoting astrocyte survival and adaptive migratory activity under ischemic‐like conditions in vitro.

### 3.10 LMP2 Shapes Astrocyte Reactive State Remodeling Through Coordinated Modulation of NF‐κB and TGF‐β1/Smad3 Signaling Under Ischemic Conditions

**FIGURE 9:**
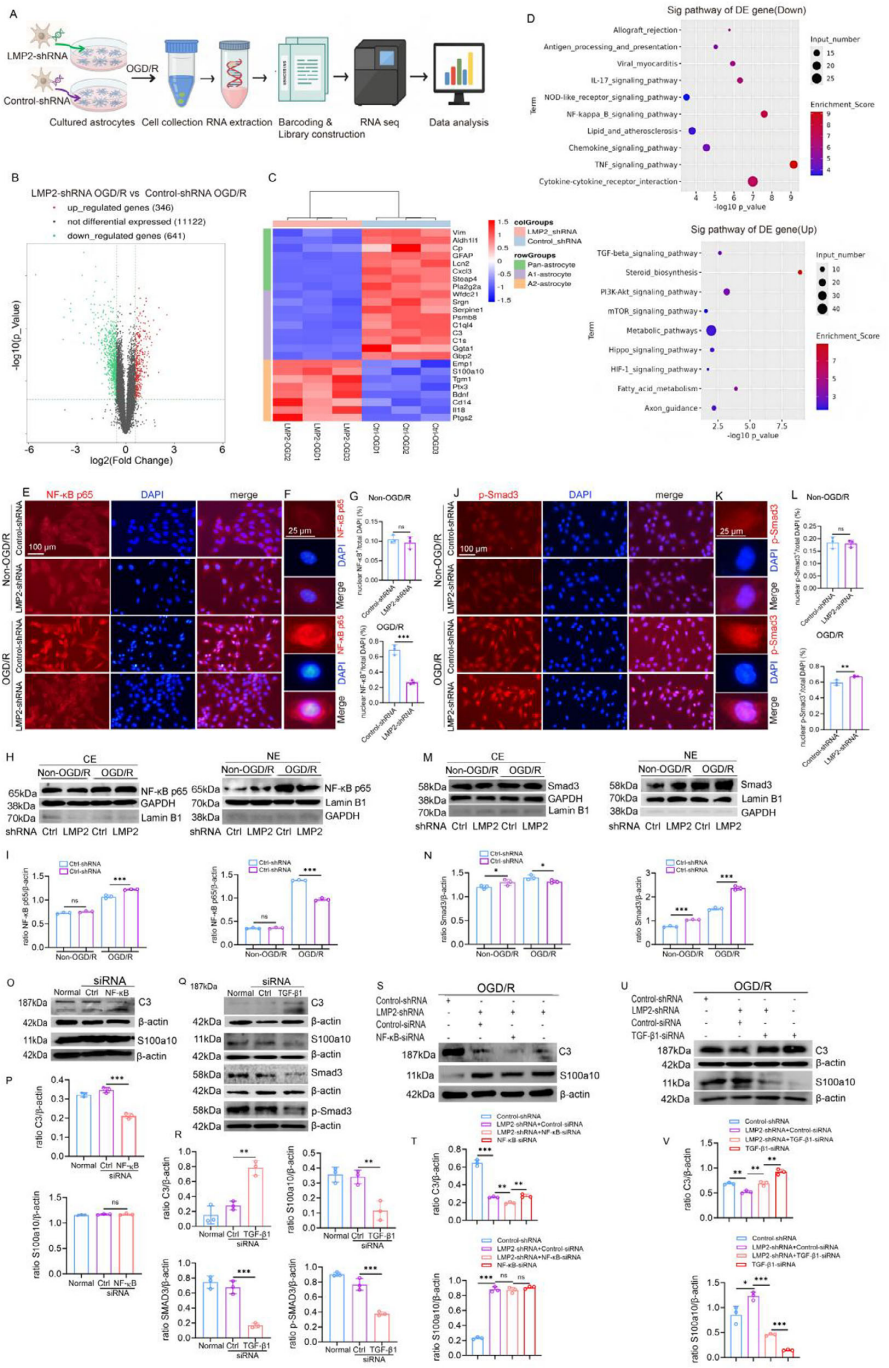
LMP2 shapes astrocyte reactive state remodeling through coordinated modulation of NF‐κB and TGF‐β1/Smad3 signaling under ischemic conditions. (A–D) RNA‐seq analysis of CTX‐TNA2 astrocytes following LMP2 knockdown under OGD/R conditions. (A) Schematic workflow of RNA sequencing. (B) Volcano plot of differentially expressed genes. (C) Unsupervised hierarchical clustering of representative differentially expressed genes. (D) KEGG pathway enrichment analysis of upregulated and downregulated genes. RNA sequencing was performed using three biologically independent CTX‐TNA2 astrocyte cultures for each experimental group (*n* = 3 biological replicates per group). (E–I) Representative immunofluorescence images, corresponding quantification, and cytoplasmic (CE) and nuclear (NE) Western blot analyses of NF‐κB p65 localization in CTX‐TNA2 astrocytes under the indicated conditions. Higher‐magnification images of the boxed regions are shown in (F), and quantification of nuclear NF‐κB p65 fluorescence intensity is shown in (G). GAPDH and Lamin B1 served as cytoplasmic and nuclear loading controls, respectively. CE, cytoplasmic extract; NE, nuclear extract. Scale bars = 100 µm (E) and 25 µm (F). (J–N) Representative immunofluorescence images, corresponding quantification, and cytoplasmic (CE) and nuclear (NE) Western blot analyses of p‐Smad3 and Smad3 localization in CTX‐TNA2 astrocytes under the indicated conditions. Higher‐magnification images of the boxed regions are shown in (K), and quantification of nuclear p‐Smad3 fluorescence intensity is shown in (L). GAPDH and Lamin B1 served as cytoplasmic and nuclear loading controls, respectively. Scale bars = 100 µm (J) and 25 µm (K). (O,P) Representative Western blot images and corresponding quantification of C3 and S100a10 protein expression following NF‐κB knockdown. Ctrl, control siRNA. (Q,R) Representative Western blot images and corresponding quantification of C3, S100a10, Smad3, and p‐Smad3 protein expression following TGF‐β1 knockdown. Ctrl, control siRNA. (S–V) Representative Western blot images and corresponding quantification of C3 and S100a10 protein expression following co‐silencing of LMP2 and NF‐κB or TGF‐β1 under OGD/R conditions. Data are presented as mean ± SD from three independent experiments using one‐way ANOVA with LSD's post hoc test. ns, not significant; ^*^ *p* < 0.05, ^**^ *p* < 0.01, ^***^ *p* < 0.001.

To further investigate the signaling networks associated with LMP2‐dependent astrocyte reactive state remodeling under ischemic conditions, RNA sequencing (RNA‐seq) was performed in CTX‐TNA2 astrocytes subjected to 2 h of oxygen–glucose deprivation followed by 24 h of reperfusion (Figure 9A). A total of 987 differentially expressed genes (DEGs) were identified between LMP2‐shRNA and control‐shRNA groups, including 346 upregulated and 641 downregulated genes, as illustrated by the volcano plot (Figure 9B). Representative heatmap analysis further revealed distinct transcriptional profiles between the two groups, with a general suppression of inflammation‐related genes and concurrent upregulation of genes associated with cellular repair, metabolic adaptation, and stress responses following LMP2 silencing (Figure 9C). KEGG pathway enrichment analysis further demonstrated that multiple immune‐inflammatory pathways, including TNF, NF‐κB, IL‐17, Toll‐like receptor, and cytokine–cytokine receptor interaction signaling, were significantly downregulated in the LMP2‐shRNA group. In contrast, pathways related to cellular adaptation and repair, such as TGF‐β signaling, fatty acid metabolism, axon guidance, and HIF‐1 signaling, were substantially enriched (Figure 9D). Collectively, these transcriptomic analyses suggest that LMP2 is associated with broad alterations in inflammatory and reparative transcriptional programs in astrocytes under ischemic stress.

Given the central roles of NF‐κB and TGF‐β1/Smad3 signaling in astrocyte reactivity [8, 37, 38], we next examined whether LMP2 modulates these pathways at the functional level. In vivo, astrocyte‐specific deletion of LMP2 suppressed NF‐κB activation while enhancing TGF‐β1/Smad3 signaling (Figure 7B). To further characterize the astrocyte‐associated signaling alterations linked to LMP2 suppression, we performed in vitro analyses in CTX‐TNA2 astrocytes. Immunofluorescence analysis revealed that OGD/R stimulation induced robust activation of NF‐κB signaling, characterized by increased nuclear translocation of NF‐κB p65 in control astrocytes. Notably, LMP2 silencing significantly attenuated NF‐κB nuclear localization, as evidenced by reduced co‐localization of NF‐κB with DAPI and a decreased proportion of nuclear NF‐κB–positive cells (Figure 9E–G). Consistently, cytoplasmic and nuclear fractionation followed by Western blot analysis demonstrated that LMP2 knockdown significantly reduced the accumulation of NF‐κB p65 in the nuclear fraction while increasing its retention in the cytoplasmic fraction under OGD/R conditions (Figure 9H,I), supporting a role for LMP2 in regulating NF‐κB nuclear translocation under ischemic‐like conditions.

In parallel, we assessed the activation status of the TGF‐β1/Smad3 pathway. OGD/R stimulation induced activation of the Smad3 pathway, whereas LMP2 silencing further enhanced both Smad3 phosphorylation and its nuclear accumulation. Immunofluorescence analysis demonstrated a marked increase in nuclear p‐Smad3 in LMP2‐silenced CTX‐TNA2 astrocytes compared with controls (Figure 9J–L), indicating enhanced activation of the TGF‐β1/Smad3 pathway. Consistently, cytoplasmic and nuclear fractionation followed by Western blot analysis demonstrated that cytoplasmic Smad3 levels were modestly decreased, whereas nuclear Smad3 accumulation was significantly increased after LMP2 knockdown (Figure 9M,N), consistent with enhanced Smad3 nuclear translocation following LMP2 silencing.

Notably, analysis of whole‐cell lysates further corroborated these findings at the pathway activation level. Western blot analysis revealed that LMP2 knockdown significantly suppressed NF‐κB signaling under OGD/R conditions, as evidenced by decreased phosphorylation of NF‐κB p65 and IκBα, accompanied by an accumulation of total IκBα (Figure S10A,B). Conversely, LMP2 silencing robustly enhanced the activation of the TGF‐β1/Smad3 pathway, as indicated by increased expression of TGF‐β1, total Smad3, and phosphorylated Smad3 (p‐Smad3) (Figure S10C,D). Overall, these findings support a role for LMP2 in coordinated regulation of NF‐κB‐associated inflammatory signaling and TGF‐β1/Smad3‐associated reparative signaling under OGD/R conditions.

To directly evaluate the functional roles of these pathways in astrocyte reactive state transitions, we performed targeted siRNA‐mediated knockdown experiments. NF‐κB knockdown (validated in Figure S11A–C) led to a pronounced reduction in C3 expression (*p* < 0.001) without significantly affecting S100a10 (*p* > 0.05) (Figure 9O,P). By comparison, TGF‐β1 silencing (validated in Figure S12A,B) prominently increased C3 expression while reducing S100a10, Smad3, and p‐Smad3 levels (*p* < 0.01, Figure 9Q,R), indicating a shift toward inflammatory astrocyte reactive states. Consistently, TGF‐β1 knockdown further amplified inflammatory responses, as evidenced by elevated TNF‐α and IL‐1β levels and increased cleaved caspase‐3 (p17), accompanied by a significant reduction in anti‐inflammatory and neurotrophic factors (IL‐10, BDNF, and GDNF) (*p* < 0.001, Figure S12C,D). These findings suggest that TGF‐β1/Smad3 signaling is associated with reparative astrocyte‐related programs while limiting inflammatory state transitions following OGD/R.

Finally, to further determine whether NF‐κB and TGF‐β1/Smad3 signaling contribute to the signaling alterations associated with LMP2 suppression on astrocyte phenotypic regulation, co‐silencing experiments were performed. Combined knockdown of LMP2 and NF‐κB resulted in the lowest C3 expression levels among all groups, further supporting the role of NF‐κB signaling in driving pro‐inflammatory astrocyte responses. In contrast, simultaneous silencing of LMP2 and TGF‐β1 largely counteracted the reparative phenotypic shift induced by LMP2 inhibition, as evidenced by increased C3 expression and reduced S100a10 expression (Figure 9S–V). These findings suggest that the effects of LMP2 on astrocyte functional reprogramming are mediated, at least in part, through coordinated modulation of NF‐κB and TGF‐β1/Smad3 pathways.

**FIGURE 10:**
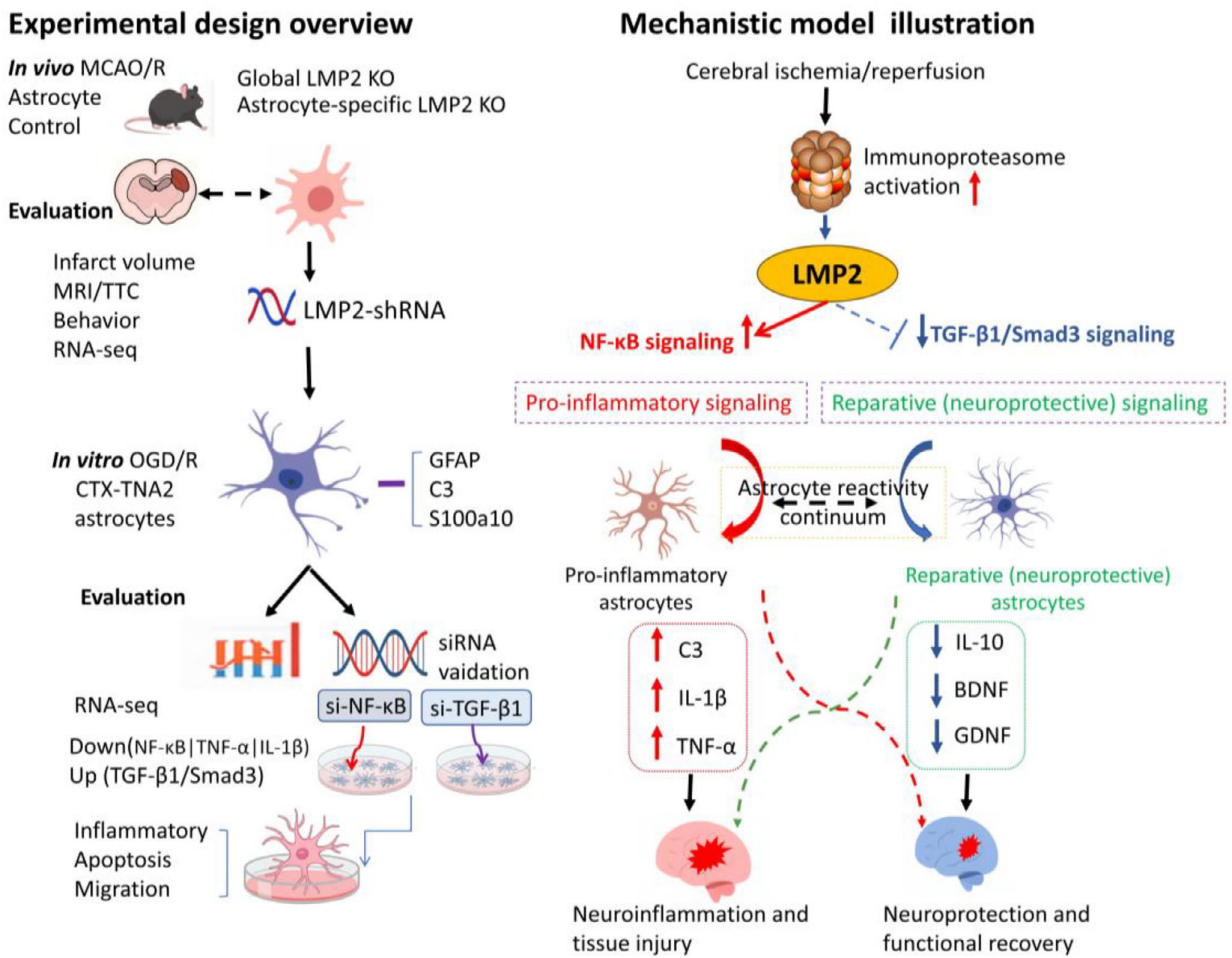
Proposed working model of astrocytic LMP2‐associated reactive state remodeling following cerebral ischemia/reperfusion. In vivo, global LMP2 knockout rats and astrocyte‐specific LMP2 conditional knockout mice were subjected to middle cerebral artery occlusion/reperfusion (MCAO/R), followed by assessment of infarct volume, MRI/TTC analysis, behavioral outcomes, and transcriptomic profiling. In vitro, oxygen‐glucose deprivation/reoxygenation (OGD/R) was applied to CTX‐TNA2 astrocytes with LMP2 knockdown, and astrocyte reactivity together with associated markers (GFAP, C3, and S100a10) was evaluated. Targeted siRNA‐mediated inhibition of NF‐κB or TGF‐β1 was further used to investigate signaling pathways involved in astrocyte functional remodeling. Under ischemic conditions, cerebral ischemia/reperfusion induced immunoproteasome activation and increased LMP2 expression, accompanied by coordinated activation of NF‐κB‐associated inflammatory signaling and suppression of TGF‐β1/Smad3‐associated reparative signaling. These signaling alterations were associated with shifts in astrocyte reactive states along the inflammatory‐reparative continuum toward more inflammatory and maladaptive programs. Conversely, LMP2 suppression attenuated inflammatory signaling, enhanced reparative and adaptive astrocyte‐associated programs, reduced neuroinflammation and tissue injury, and improved neurological recovery following ischemic stroke.

Taken together, these findings support a role for LMP2 in astrocyte reactive state remodeling along the inflammatory–reparative continuum under ischemic conditions. Mechanistically, LMP2 appears to coordinately modulate NF‐κB‐associated inflammatory signaling and TGF‐β1/Smad3‐associated reparative signaling, thereby influencing astrocyte functional remodeling following cerebral ischemia/reperfusion injury (Figure 10).

## 4 Discussion

This study identifies astrocytic LMP2 as a potential regulator of post‐ischemic neuroinflammation and astrocyte functional remodeling following cerebral ischemia/reperfusion injury. Using both global and astrocyte‐specific knockout models, we demonstrate that LMP2 deficiency markedly attenuates infarct volume, improves neurological recovery, and shifts astrocyte responses toward a less inflammatory and more reparative state. Mechanistically, LMP2 is associated with enhanced NF‐κB‐dependent inflammatory signaling and suppression of TGF‐β1/Smad3‐associated reparative pathways following ischemic injury. Importantly, our transcriptomic, single‐cell, and functional analyses support a model in which reactive astrocytes do not exist as rigid binary phenotypes, but instead undergo dynamic and context‐dependent transitions along an inflammatory‐reparative continuum. Together, these findings support a model in which LMP2 may contribute to astrocyte state plasticity and neuroinflammatory remodeling following cerebral ischemia/reperfusion injury.

Previous studies have largely attributed immunoproteasome activity to microglia and infiltrating leukocytes [39, 40]. Here, we extend this paradigm by demonstrating an important role for LMP2 in astrocytes during ischemic brain injury. Following MCAO/R, LMP2 expression was markedly induced in GFAP^+^ astrocytes and coincided with robust activation of inflammatory signaling pathways and increased production of pro‐inflammatory mediators, including TNF‐α, IL‐1β, and CXCL10. In contrast, genetic deletion of LMP2 significantly attenuated these inflammatory responses while enhancing anti‐inflammatory and neurotrophic factors such as IL‐10, BDNF, and GDNF, indicating that LMP2 functions not merely as a downstream stress‐responsive molecule, but as an active regulator of astrocyte‐mediated inflammatory remodeling under ischemic conditions. Notably, our analysis of additional immunoproteasome subunits, including LMP7 and MECL‐1, as well as constitutive proteasome components, did not reveal compensatory upregulation following LMP2 deletion. This suggests that the observed effects are not simply attributable to global alterations in proteasome composition, but instead reflect a functionally specific role of LMP2 within astrocytes under ischemic conditions. These findings are consistent with previous reports demonstrating that pharmacological inhibition of immunoproteasome subunits mitigates CNS inflammation in models of experimental autoimmune encephalomyelitis and traumatic brain injury [41, 42].

Astrocytes are highly responsive to oxidative and inflammatory stress and require rapid proteostatic adaptation to maintain cellular homeostasis following ischemic injury [34]. Unlike constitutive proteasomes, immunoproteasomes are preferentially induced under inflammatory conditions and possess specialized catalytic properties that facilitate the degradation of oxidized or damaged proteins [20]. Because reactive astrocytes undergo profound metabolic, secretory, and transcriptional remodeling after ischemic injury [26, 43, 44], efficient proteostatic adaptation may be particularly important for maintaining astrocyte functional plasticity under inflammatory stress. In this context, ischemia‐induced upregulation of astrocytic LMP2 may represent a stress‐adaptive proteostatic mechanism that secondarily reshapes inflammatory signaling networks and astrocyte functional states. Interestingly, astrocyte‐specific deletion of LMP2 also attenuated microglial activation following MCAO/R, suggesting that LMP2‐dependent astrocytic signaling exerts secondary regulatory effects on neighboring glial populations. This observation aligns with the well‐established concept of astrocyte‐microglia crosstalk mediated through complement and chemokine signaling [45, 46]. Such intercellular communication is critical for coordinating neuroinflammatory responses, as excessive microglial activation exacerbates neuronal injury, whereas balanced glial interactions support debris clearance and tissue repair [47, 48]. Together, these findings position astrocytic LMP2 as a potential regulator of glial network dynamics during post‐ischemic neuroinflammation. Nevertheless, because immunoproteasome activation also occurs in microglia, endothelial cells, and infiltrating immune populations after stroke, we cannot completely exclude the contribution of non‐astrocytic cell populations to the overall neuroprotective phenotype observed in vivo.

Mechanistically, our data indicate that LMP2 is closely associated with coordinated regulation of the NF‐κB and TGF‐β1/Smad3 signaling pathways, two central signaling axes implicated in astrocyte inflammatory and reparative responses. NF‐κB signaling represents a canonical pro‐inflammatory pathway whose activation depends on phosphorylation and proteasomal degradation of the inhibitory protein IκBα [49]. In the present study, LMP2 deficiency consistently reduced NF‐κB p65 phosphorylation, impaired nuclear translocation, and increased cytoplasmic retention of NF‐κB p65, accompanied by suppression of inflammatory mediators including C3, IL‐1β, and MMP9. Notably, our additional findings further demonstrated that LMP2 silencing increased total IκBα levels while reducing phosphorylation of IκBα under OGD/R conditions, suggesting that LMP2 may regulate NF‐κB activation, at least in part, through modulation of upstream inhibitory components such as IκBα. In parallel, LMP2 deletion enhanced activation of the TGF‐β1/Smad3 signaling pathway, as evidenced by increased Smad3 phosphorylation and enhanced nuclear accumulation of Smad3 and p‐Smad3. Given the established role of TGF‐β1/Smad3 signaling in promoting neuroprotective and reparative programs [50, 51, 52], these findings suggest that LMP2 biases astrocyte responses toward inflammatory programs while constraining reparative signaling pathways. Importantly, our data do not support a direct substrate‐specific mechanism whereby LMP2 selectively targets individual signaling proteins for proteasomal degradation. Instead, the observed effects are more consistent with pathway‐level regulation in which LMP2, as a catalytic component of the immunoproteasome, modulates cellular proteostasis and thereby influences stress‐responsive signaling network activity in a context‐dependent manner. Within this framework, LMP2 may regulate the turnover or activation of upstream signaling regulators, ultimately leading to coordinated changes in downstream inflammatory and reparative pathways. For example, inhibitory regulators such as Smad7 have been reported to undergo proteasome‐dependent turnover and thereby modulate TGF‐β pathway activity [53, 54]. Although we did not directly examine Smad7 in the present study, our findings demonstrate that LMP2 impacts TGF‐β1/Smad3 signaling at multiple levels, including expression, phosphorylation, and nuclear translocation.

Consistent with these mechanistic observations, siRNA‐mediated perturbation experiments demonstrated that NF‐κB inhibition selectively attenuated pro‐inflammatory astrocyte responses, whereas TGF‐β1 silencing shifted astrocytes toward a more inflammatory and less reparative state. Furthermore, combined silencing experiments suggested that the effects of LMP2 on astrocyte functional remodeling are mediated, at least in part, through coordinated modulation of NF‐κB and TGF‐β1/Smad3 signaling pathways. Together, these findings support a model in which LMP2 may regulates astrocyte functional state transitions at the signaling‐network level rather than through control of a single downstream substrate. These findings collectively suggest that astrocyte functional states may be determined not by activation of a single pathway alone, but rather by the relative balance between inflammatory and reparative signaling networks under ischemic stress.

Beyond these mechanistic findings, our study further supports the emerging concept that reactive astrocytes exist along a dynamic spectrum of transcriptional states rather than rigid binary phenotypes. Recent single‐cell transcriptomic studies have increasingly challenged the traditional A1/A2 framework and instead support a continuum model of astrocyte reactivity [7]. Consistent with this concept, our single‐cell analyses revealed that astrocyte‐associated markers such as C3 and S100a10 were broadly distributed across multiple astrocyte subclusters rather than being restricted to mutually exclusive populations. These observations suggest that such markers likely represent overlapping functional features distributed across heterogeneous reactive states instead of defining discrete astrocyte phenotypes. Within this framework, LMP2 appears to function as a regulatory node that biases astrocyte state distribution toward inflammatory or reparative trajectories through coordinated modulation of stress‐responsive signaling pathways.

This conceptual framework may also help explain the limited translational success of previous therapeutic strategies aimed at broadly suppressing astrocyte activation after stroke. Indiscriminate inhibition of astrocyte reactivity may simultaneously impair adaptive neuroprotective and tissue‐repair functions that are essential for neurological recovery. In contrast, selective modulation of astrocyte state plasticity may provide a more balanced therapeutic strategy by limiting detrimental neuroinflammatory responses while preserving endogenous repair programs after ischemic injury. More broadly, these findings support the emerging concept that selective regulation of glial state plasticity, rather than nonspecific suppression of neuroinflammation, may represent a more effective strategy for post‐stroke immune remodeling and precision neuroimmunomodulation.

From a translational perspective, selective targeting of LMP2 may therefore represent a mechanistically informed therapeutic strategy distinct from broad immunosuppression or pan‐proteasome inhibition. In contrast to constitutive proteasome inhibition, which can disrupt fundamental cellular proteostasis [55], selective immunoproteasome inhibitors such as ONX‐0914 and DPLG3 have demonstrated anti‐inflammatory efficacy in several preclinical disease models [16, 19, 56]. However, several important translational challenges should be acknowledged. The ability of currently available immunoproteasome inhibitors to efficiently penetrate the blood–brain barrier and achieve therapeutically relevant concentrations within ischemic brain tissue remains incompletely understood. In addition, systemic administration is unlikely to confer astrocyte‐specific targeting and may simultaneously affect peripheral immune responses, which is particularly relevant in stroke patients already vulnerable to post‐stroke immunosuppression and infection. Furthermore, the selectivity of immunoproteasome inhibitors is often dose‐dependent, and higher concentrations may reduce subunit specificity and inadvertently inhibit constitutive proteasome activity. Therefore, although our findings support astrocytic LMP2 as a promising therapeutic target, they should currently be interpreted as proof‐of‐concept rather than direct evidence of immediate pharmacological feasibility. Future development of brain‐penetrant and astrocyte‐targeted delivery strategies, including nanoparticle‐based approaches, may further improve the therapeutic specificity and translational potential of immunoproteasome modulation in ischemic stroke.

Beyond experimental models, the clinical relevance of immunoproteasome activation in human stroke also warrants consideration. In our previous clinical studies, elevated plasma levels of LMP2/immunoproteasome were associated with an increased risk of hemorrhagic transformation in patients with acute ischemic stroke [57] and were predictive of poor functional outcomes and post‐stroke cognitive impairment at 90 days [58]. These findings suggest that immunoproteasome activation is clinically relevant in human cerebrovascular disease. Nevertheless, these observations were derived from peripheral blood analyses and do not directly reflect astrocytic LMP2 expression within the ischemic brain. Whether astrocytic LMP2 undergoes similar functional remodeling in human post‐ischemic tissue remains unknown. Future studies integrating postmortem human brain tissue, spatial transcriptomics, and single‐cell approaches will therefore be important for validating the translational relevance of the astrocytic LMP2 regulatory axis identified in the present study.

Several limitations should be acknowledged. First, the present study primarily focused on the acute to subacute stages following cerebral ischemia/reperfusion, with mechanistic and transcriptomic analyses centered on the 72 h timepoint corresponding to peak astrocyte activation. Although this window captures critical early neuroinflammatory events, astrocyte responses continue to evolve during later stages of injury and recovery. Future studies incorporating longer observation periods and multi‐timepoint analyses will therefore be necessary to define the long‐term impact of LMP2 modulation on chronic brain remodeling and functional recovery. Second, although our single‐cell analyses provide important insights into astrocyte heterogeneity, the current study lacks spatially resolved transcriptomic information, and future integration of spatial transcriptomics may further clarify region‐specific astrocyte responses within the ischemic microenvironment. Third, although GFAP‐Cre models predominantly target astrocytes, potential off‐target recombination cannot be completely excluded. In addition, although CTX‐TNA2 astrocytic cells provide a reproducible and experimentally controllable in vitro system for mechanistic investigation, they are derived from neonatal rat cortex and therefore may not fully recapitulate the signaling properties and stress responses of mature adult astrocytes in the ischemic brain. In particular, developmental differences may influence immunoproteasome regulation, NF‐κB activation, and TGF‐β responsiveness under ischemic conditions. Therefore, although the current in vitro findings provide mechanistic support for the in vivo observations, further validation in adult primary astrocytes and more physiologically relevant models will be important for strengthening the cell‐autonomous and translational interpretation of our findings. Only male animals were included in the present study, and whether sex‐dependent differences influence astrocyte immunoproteasome responses after ischemic injury warrants further investigation. Furthermore, although assessment of proteasome catalytic activities may provide additional mechanistic insights, bulk measurements from whole‐brain lysates would not readily distinguish astrocyte‐specific effects because of the substantial cellular heterogeneity within ischemic tissue. Finally, the precise molecular intermediates linking LMP2 to NF‐κB and TGF‐β1/Smad3 signaling remain incompletely defined and will require future investigation using co‐immunoprecipitation, ubiquitination assays, and substrate‐resolved proteasome analyses.

## 5 Conclusion

In summary, our findings support a functional role for astrocytic LMP2 in inflammatory‐reparative signaling remodeling following cerebral ischemia/reperfusion injury. LMP2 suppression attenuated NF‐κB‐associated inflammatory signaling while enhancing TGF‐β1/Smad3‐associated reparative responses, accompanied by shifts in astrocyte functional states toward less inflammatory and more adaptive programs. Importantly, our transcriptomic and single‐cell analyses further support the concept that reactive astrocytes exist along a dynamic continuum of context‐dependent states rather than rigid binary phenotypes. More broadly, these findings expand current understanding of immunoproteasome biology beyond classical immune regulation and identify astrocytes as important immunoproteasome‐responsive regulators of neuroinflammatory state plasticity in the injured CNS. Collectively, our study establishes a conceptual and mechanistic framework linking astrocytic LMP2 with reactive state remodeling following stroke and highlights LMP2 as a promising therapeutic target for precision modulation of post‐ischemic neuroinflammatory responses.

## Author Contributions

Yanguang Mao, Rulan Ma, Zejing Lin contributed equally to this work. Yanguang Mao, Rulan Ma, Zejing Lin, Huiying Zhao, Yuexian Liu, and Yonghe Su completed experiments(investigation) and analyzed the data. Xu Zhang reviewed the manuscript. Xingyong Chen: Writing – review & editing, Writing – original draft, visualization, validation, supervision, resources, project administration, funding acquisition, formal analysis, data curation, conceptualization. All authors reviewed and approved the manuscript.

## Conflicts of Interest

The authors declare no conflicts of interest.

## Supporting information




**Supporting File 1**: advs76807‐sup‐0001‐SuppMat.docx.

**Supporting File 2**: advs76807‐sup‐0002‐TablesS1.docx.

## Data Availability

The data that support the findings of this study are available from the corresponding author upon reasonable request.
