# Supplementary material for: Astrocytic LMP2 Coordinates NF‐κB and TGF‐β1/Smad3 Signaling to Drive Neuroinflammation after Cerebral Ischemia/Reperfusion
Source: Adv Sci (Weinh). 2026 Jul 27:e23902. Online ahead of print. doi: 10.1002/advs.202523902 (PMC13403376; doi:10.1002/advs.202523902)
Supplement: Supplementary file 1 — Supporting File 1: advs76807‐sup‐0001‐SuppMat.docx. [file ADVS-9999-e23902-s001.docx]

**Supplementary Figures and Legends**

**
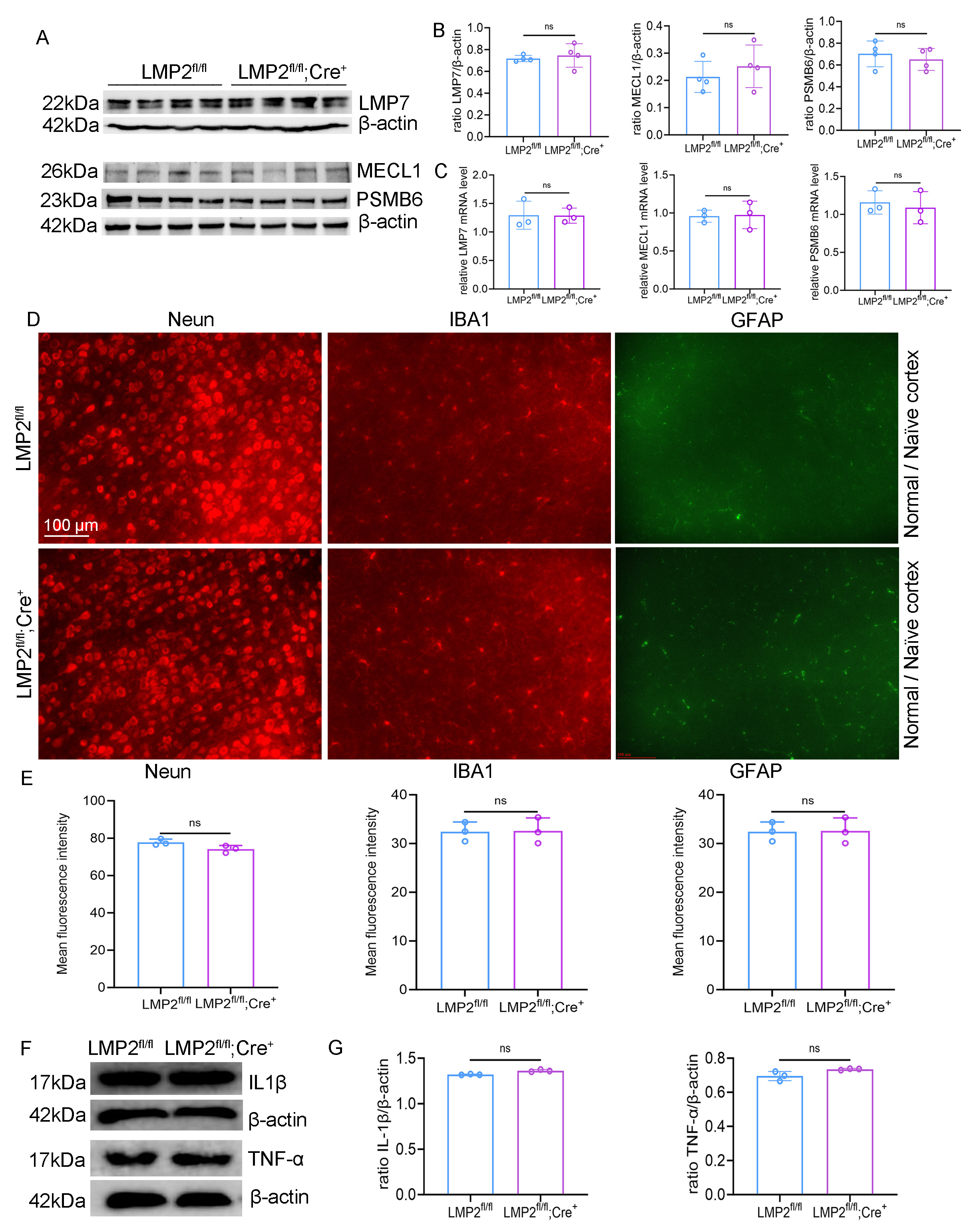
**

**Supplementary Figure 1. Astrocyte-specific LMP2 deletion does not alter basal proteasome subunit expression, neural cell markers, or inflammatory protein expression under physiological conditions.** (A-B) Representative Western blot images and corresponding quantification of LMP7, MECL-1, and PSMB6 protein expression in the cerebral cortex of LMP2^fl/fl^ and LMP2^fl/fl^;Cre^+^ mice under physiological conditions. n = 4 mice per group. Data are presented as mean ± SD from three independent experiments using unpaired two-tailed Student’s t test. (C) Relative mRNA expression levels of Psmb8 (LMP7), Psmb10 (MECL-1), and Psmb6 measured by RT-qPCR. Data are presented as mean ± SD from three independent experiments using unpaired two-tailed Student’s t test. (D-E) Representative immunofluorescence images of NeuN, IBA1, and GFAP staining in the cerebral cortex of LMP2^fl/fl^ and LMP2^fl/fl^;Cre^+^ mice under physiological conditions. Scale bar = 100 μm. (E) Corresponding quantification of mean fluorescence intensity shown in (D). (F-G) Representative Western blot images and corresponding quantification of IL-1β and TNF-α protein expression in the cerebral cortex of LMP2^fl/fl^ and LMP2^fl/fl^;Cre^+^ mice under physiological conditions. n = 3 mice per group. Data are presented as mean ± SD from three independent experiments using unpaired two-tailed Student’s t test. ns, not significant.

**
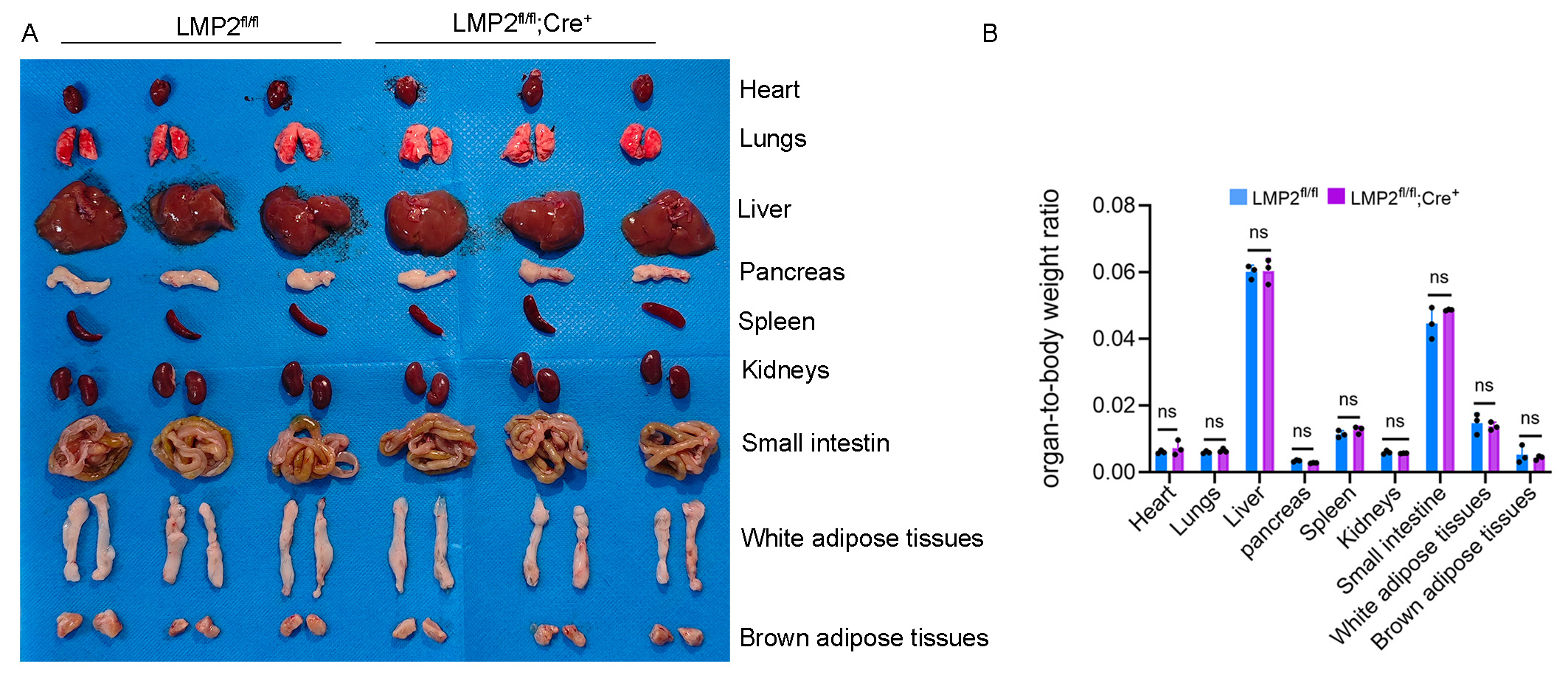
**

**Supplementary Figure 2. Gross morphology and organ-to-body weight ratios of major organs in LMP2^fl/fl^ and LMP2^fl/fl^;Cre⁺ mice. (A) Representative images of the gross morphology of the heart, lungs, liver, pancreas, spleen, kidneys, small intestine, white adipose tissue, and brown adipose tissue from LMP2^fl/fl^ and LMP2^fl/fl^;Cre⁺mice. (B) Quantification of organ-to-body weight ratios for the indicated organs and tissues.** n = 3 mice per group. Data are presented as mean ± SD from three independent experiments using unpaired two-tailed Student’s t test. ns, not significant.

**
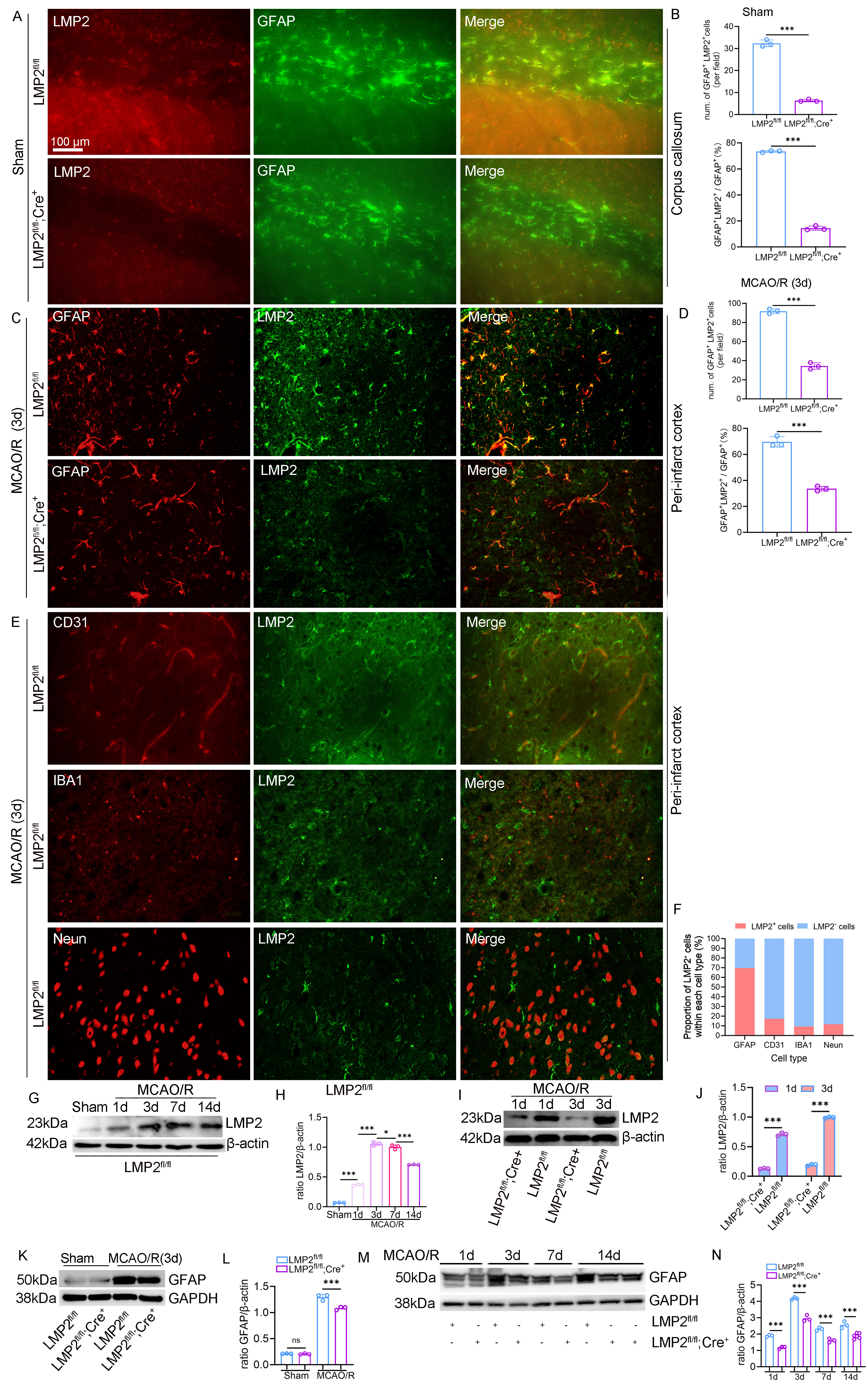
**

**Supplementary Figure 3. Cell-type-specific localization and temporal dynamics of LMP2 expression and astrocyte activation following cerebral ischemia.** (A-B) Representative immunofluorescence images and quantification of LMP2 (red) and GFAP (green) in the corpus callosum of sham LMP2^fl/fl^ and LMP2^fl/fl^;Cre⁺ mice. Quantification in (B) shows the number of GFAP⁺/LMP2⁺ cells and the percentage of GFAP⁺ cells expressing LMP2. n = 3 mice per group. Data are presented as mean ± SD from three independent experiments using unpaired two-tailed Student’s t test. (C-D) Representative immunofluorescence images and quantification of GFAP (red) and LMP2 (green) staining in the peri-infarct cortex at 3 days after MCAO/R. Quantification in (D) shows the number of GFAP⁺/LMP2⁺ cells and the percentage of GFAP⁺ cells expressing LMP2. n = 3 mice per group. Data are presented as mean ± SD from three independent experiments using unpaired two-tailed Student’s t test. (E-F) Representative immunofluorescence images showing LMP2 (green) co-staining with CD31 (endothelial cells), IBA1 (microglia), and NeuN (neurons) in the peri-infarct cortex at 3 days after MCAO/R. Quantification in (F) shows the percentage of LMP2-positive and LMP2-negative cells within each indicated cell type. (G-H) Representative Western blot images and quantification of LMP2 protein expression in the cortex and striatum at the indicated time points after MCAO/R. (I-J) Representative Western blot images and quantification of LMP2 protein expression in the peri-infarct cortex and striatum of LMP2^fl/fl^ and LMP2^fl/fl^;Cre⁺ mice at 1 and 3 days after MCAO/R. (K-L) Representative Western blot images and quantification of GFAP protein expression in the cortex and striatum under sham conditions and at 3 days after MCAO/R in LMP2^fl/fl^ and LMP2^fl/fl^;Cre⁺ mice. (M-N) Representative Western blot images and quantification of GFAP protein expression in the peri-infarct cortex and striatum at 1, 3, 7, and 14 days after MCAO/R in LMP2^fl/fl^ and LMP2^fl/fl^;Cre⁺ mice. Scale bar = 100 μm. n = 3 mice per group. Data are presented as mean ± SD from three independent experiments using one-way ANOVA with LSD’s post hoc test. ns, not significant. ^*^*P* < 0.05, ^**^*P* < 0.01, ^***^*P* < 0.001.

**
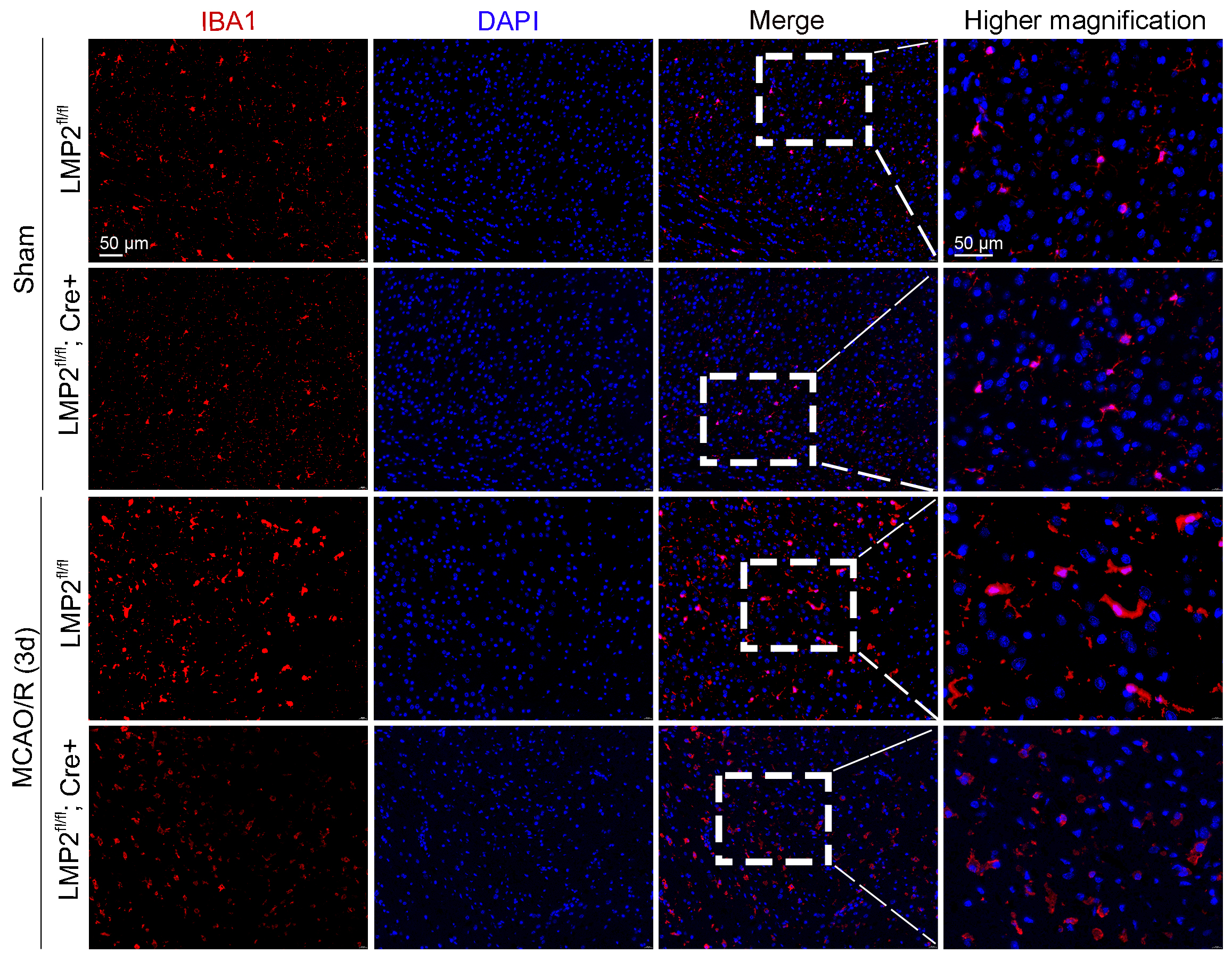
**

**Supplementary Figure 4. Astrocyte-specific deletion of LMP2 reduces IBA1 immunoreactivity following cerebral ischemia.**Representative immunofluorescence images showing IBA1 (red) and DAPI (blue) staining in the peri-infarct cortex of LMP2^fl/fl^ and LMP2^fl/fl^;Cre⁺ mice under sham conditions and at 3 days after MCAO/R. Dashed boxes indicate the regions shown at higher magnification. Scale bars = 50 μm. Data are presented from n = 3 mice per group.


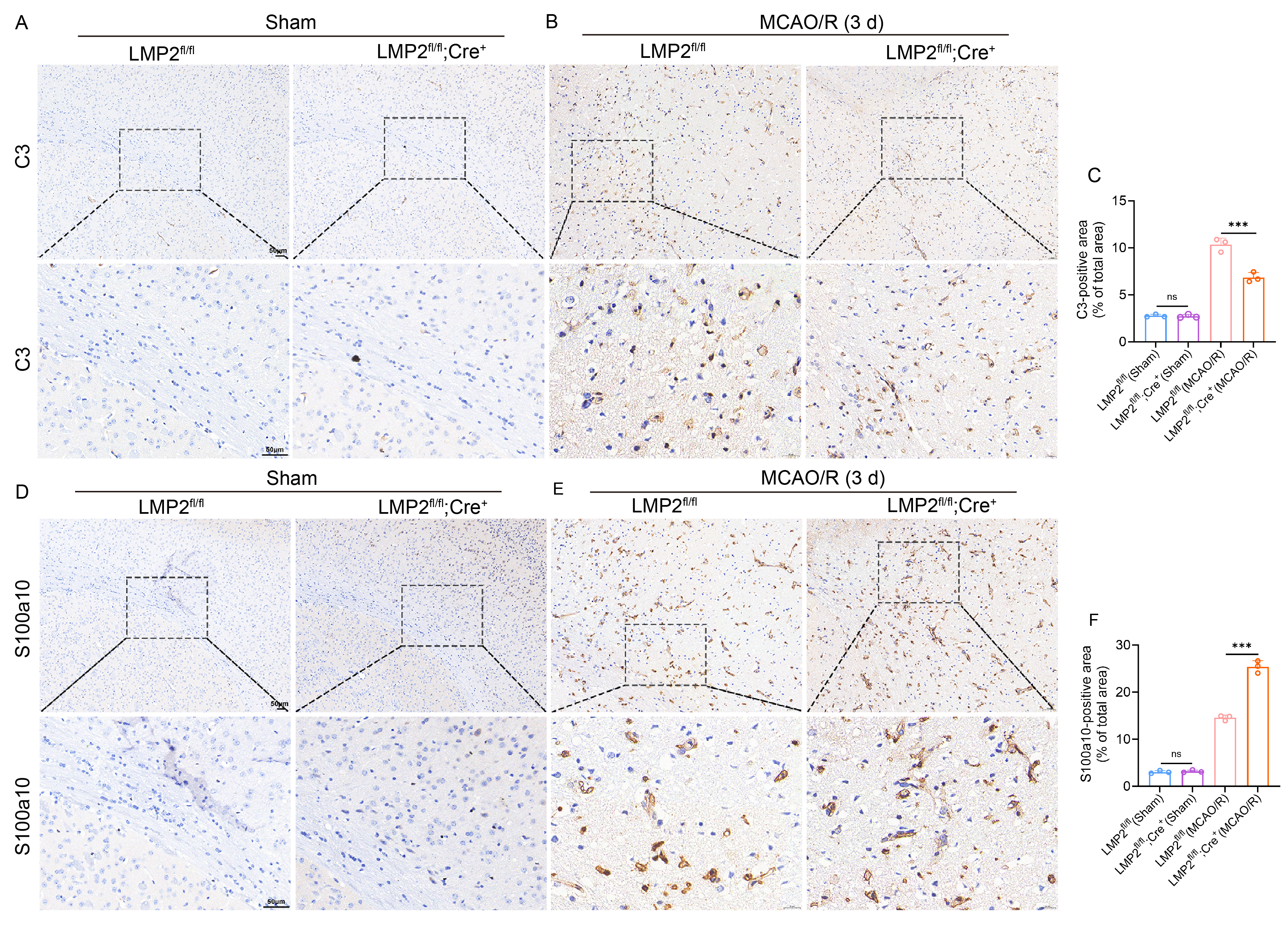


**Supplementary Figure 5. Immunohistochemical staining of C3 and S100a10 in the peri-infarct cortex and striatum following cerebral ischemia.** (A-C) Representative DAB-based immunohistochemical staining and quantification of C3 in LMP2^fl/fl^ and LMP2^fl/fl^;Cre⁺ mice under sham conditions and at 3 days after MCAO/R. Quantification in (C) shows the percentage of C3-positive area. (D-F) Representative DAB-based immunohistochemical staining and quantification of S100a10 in LMP2^fl/fl^ and LMP2^fl/fl^;Cre⁺ mice under sham conditions and at 3 days after MCAO/R. Quantification in (F) shows the percentage of S100a10-positive area. Dashed boxes indicate the regions shown at higher magnification. Scale bars = 50 μm. n = 3 mice per group. Data are presented as mean ± SD from three independent experiments using one-way ANOVA with LSD’s post hoc test. ns, not significant. ^***^*P* < 0.001.

**
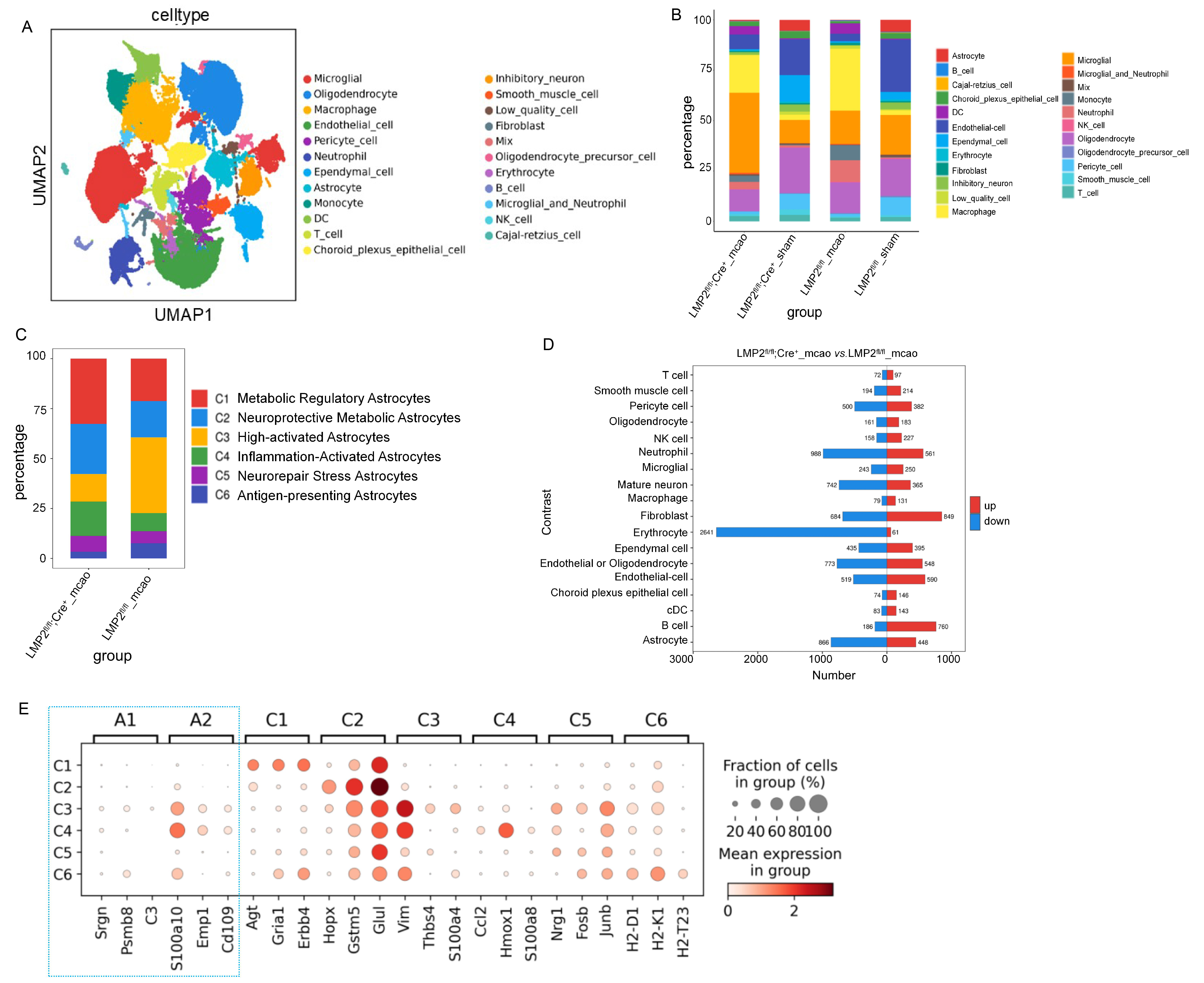
**

**Supplementary Figure 6. Single-cell RNA sequencing analysis of brain cell populations and astrocyte subclusters following cerebral ischemia.** (A) UMAP visualization of major brain cell populations identified by single-cell RNA sequencing from the peri-infarct cortex and striatum at 3 days after MCAO/R. (B) Stacked bar plot showing the relative proportions of major cell populations across the indicated experimental groups. (C) Astrocyte subclustering analysis identifying six astrocyte subclusters (C1-C6) based on transcriptomic profiles. Stacked bar plots show the relative proportions of each astrocyte subcluster in the indicated MCAO/R groups. (D) Differentially expressed genes identified in major cell populations between LMP2^fl/fl^;Cre⁺ MCAO/R and LMP2^fl/fl^ MCAO/R groups. (E) Dot plot showing the expression of representative marker genes across astrocyte subclusters (C1-C6). Dot size indicates the fraction of cells expressing each gene, and color intensity represents the mean expression level. Dashed boxes highlight representative astrocyte marker genes, including C3 and S100a10. Single-cell RNA sequencing data were generated from n = 3 biologically independent mice per group.


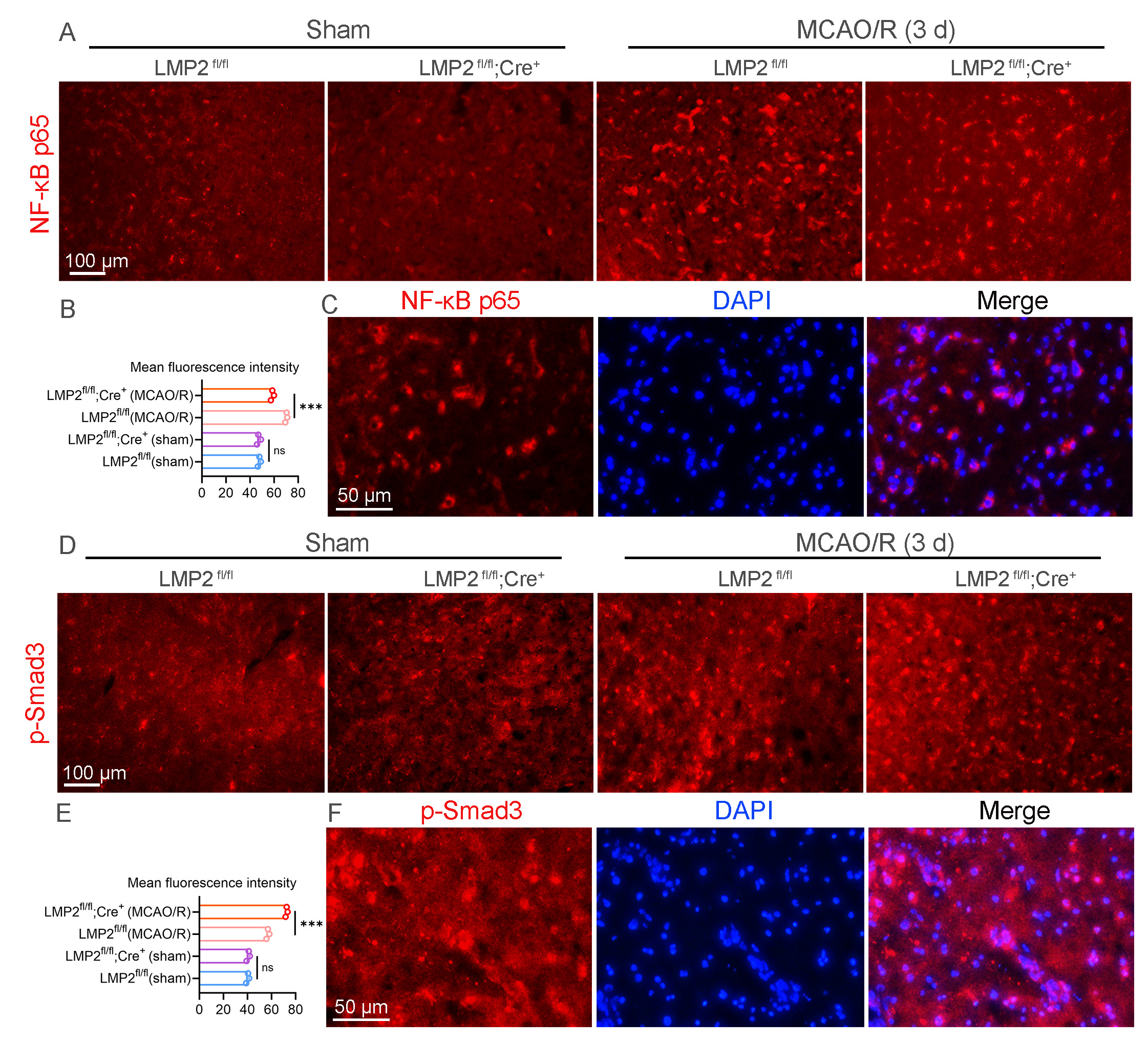


**Supplementary Figure 7. Astrocyte-specific LMP2 deletion modulates NF-κB p65 and p-Smad3 immunoreactivity following MCAO/R.** (A-B) Representative immunofluorescence images and quantification of NF-κB p65 immunoreactivity in the peri-infarct cortex of LMP2^fl/fl^ and LMP2^fl/fl^;Cre⁺ mice under sham and MCAO/R (3 d) conditions. Mean fluorescence intensity of NF-κB p65 is shown in (B). Scale bars = 100 μm. n = 3 mice per group. (C) Representative higher-magnification images of NF-κB p65 (red), DAPI (blue), and merged images showing nuclear localization of NF-κB p65 in the peri-infarct cortex following MCAO/R. Scale bars = 50 μm. (D-E) Representative immunofluorescence images and quantification of phosphorylated Smad3 (p-Smad3) immunoreactivity in the peri-infarct cortex of LMP2^fl/fl^ and LMP2^fl/fl^;Cre⁺ mice under sham and MCAO/R (3 d) conditions. Mean fluorescence intensity of p-Smad3 is shown in (E). Scale bars = 100 μm. n = 3 mice per group. (F) Representative higher-magnification images of p-Smad3 (red), DAPI (blue), and merged images showing nuclear localization of p-Smad3 in the peri-infarct cortex following MCAO/R. Scale bars = 50 μm. n = 3 mice per group. Data are presented as mean ± SD from three independent experiments using one-way ANOVA with LSD’s post hoc test. ns, not significant; ^*^*P* < 0.05, ^**^*P* < 0.01, ^***^*P* < 0.001.


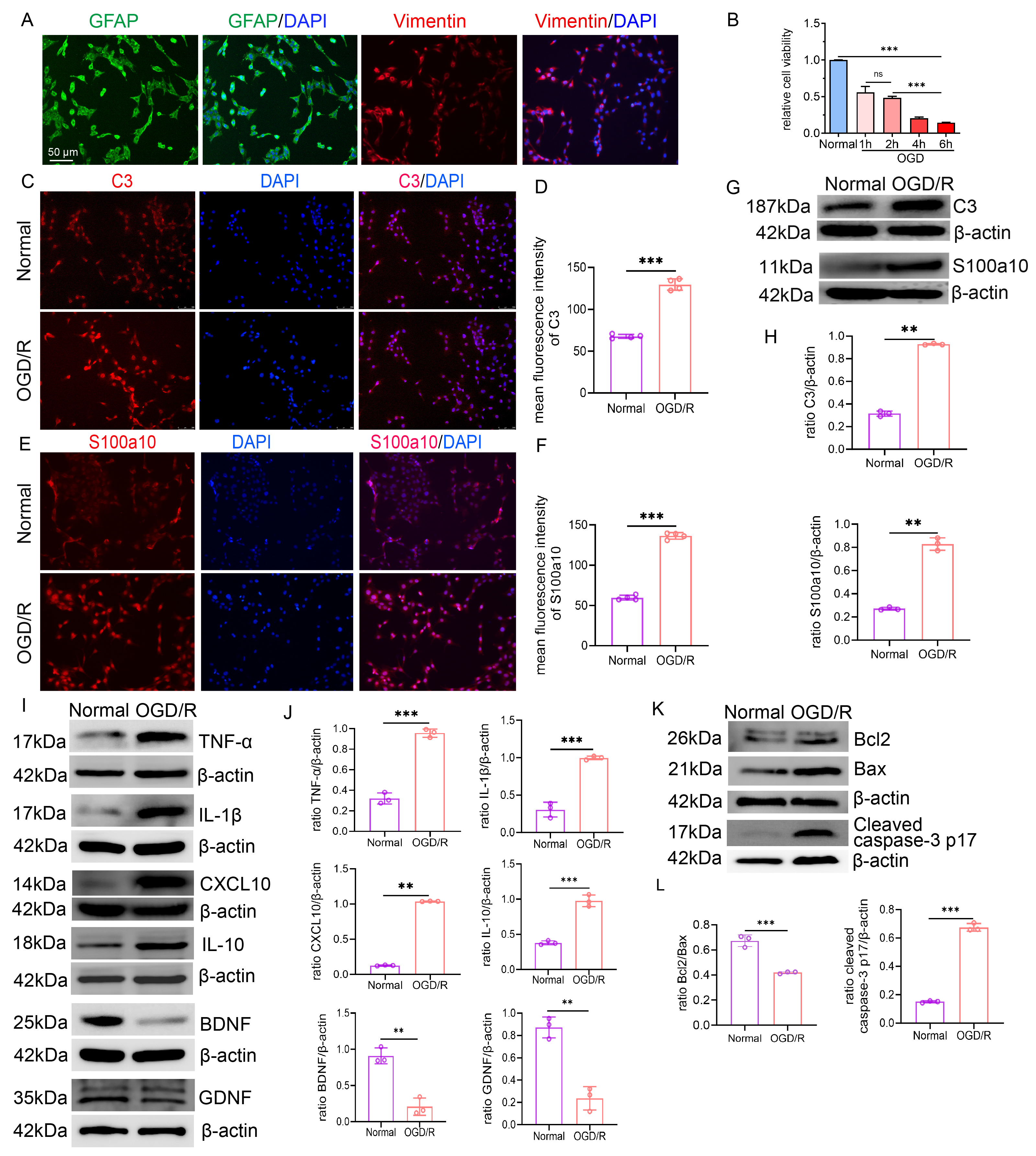


**Supplementary Figure 8. Characterization of CTX-TNA2 astrocytes and their responses to OGD/R.** (A) Representative immunofluorescence images showing GFAP (green), Vimentin (red), and DAPI (blue) staining in CTX-TNA2 astrocytes. (B) Cell viability measured by CCK-8 assay following different durations of oxygen–glucose deprivation (OGD). Cell viability was normalized to the Normal group. (C-F) Representative immunofluorescence images and quantification of C3 and S100a10 fluorescence intensity in CTX-TNA2 astrocytes under Normal and OGD/R conditions. (G-H) Representative Western blot images and quantification of C3 and S100a10 protein expression under Normal and OGD/R conditions. (I-J) Representative Western blot images and quantification of TNF-α, IL-1β, CXCL10, IL-10, BDNF, and GDNF protein expression under Normal and OGD/R conditions. (K-L) Representative Western blot images and quantification of apoptosis-related proteins, including Bcl-2, Bax, cleaved caspase-3 p17, the Bcl-2/Bax ratio, and cleaved caspase-3 p17 protein expression under Normal and OGD/R conditions. Scale bars = 50 μm. Data are presented as mean ± SD from three independent experiments using unpaired two-tailed Student’s t test. ns, not significant; ^*^*P* < 0.05, ^**^*P* < 0.01, ^***^*P* < 0.001.


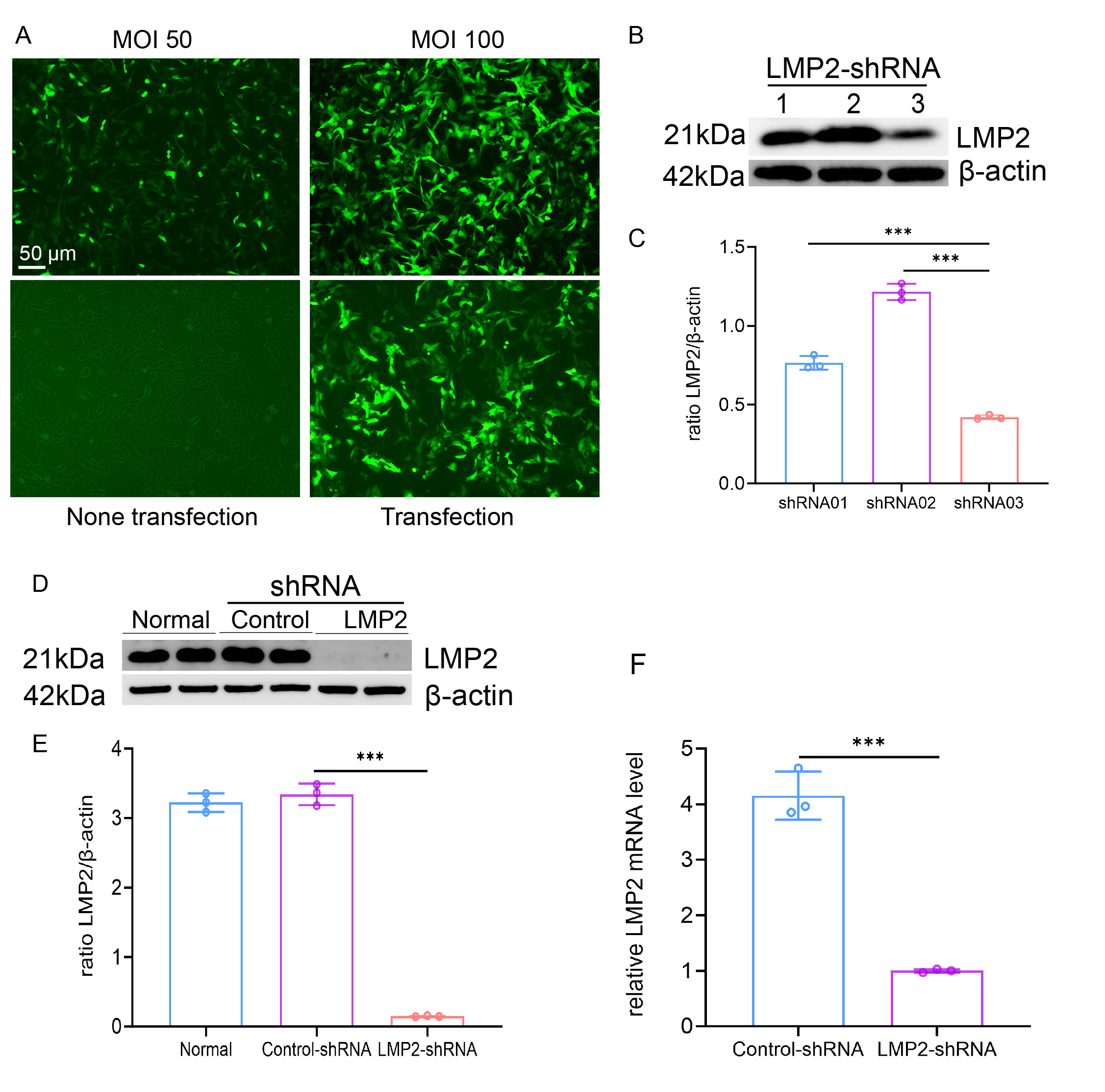


**Supplementary Figure 9. Optimization and validation of LMP2 knockdown in CTX-TNA2 astrocytes.** (A) Representative GFP fluorescence images showing lentiviral transduction efficiency in CTX-TNA2 astrocytes at multiplicities of infection (MOI) of 50 and 100. Non-transduced cells served as controls. Scale bars = 50 μm. (B-C) Representative Western blot images and quantification of LMP2 protein expression in CTX-TNA2 astrocytes transduced with three LMP2-targeting shRNAs (shRNA-1 to shRNA-3). Data are presented as mean ± SD from three independent experiments using one-way ANOVA with LSD’s post hoc test. ^***^*P* < 0.001. (D-E) Representative Western blot images and quantification of LMP2 protein expression in the Normal, Control-shRNA, and LMP2-shRNA groups. Data are presented as mean ± SD from three independent experiments using one-way ANOVA with LSD’s post hoc test. ^***^*P* < 0.001. (F) Relative LMP2 mRNA expression measured by RT-qPCR in the Control-shRNA and LMP2-shRNA groups. Data are presented as mean ± SD from three independent experiments using unpaired two-tailed Student’s t test. ^***^*P* < 0.001.

**
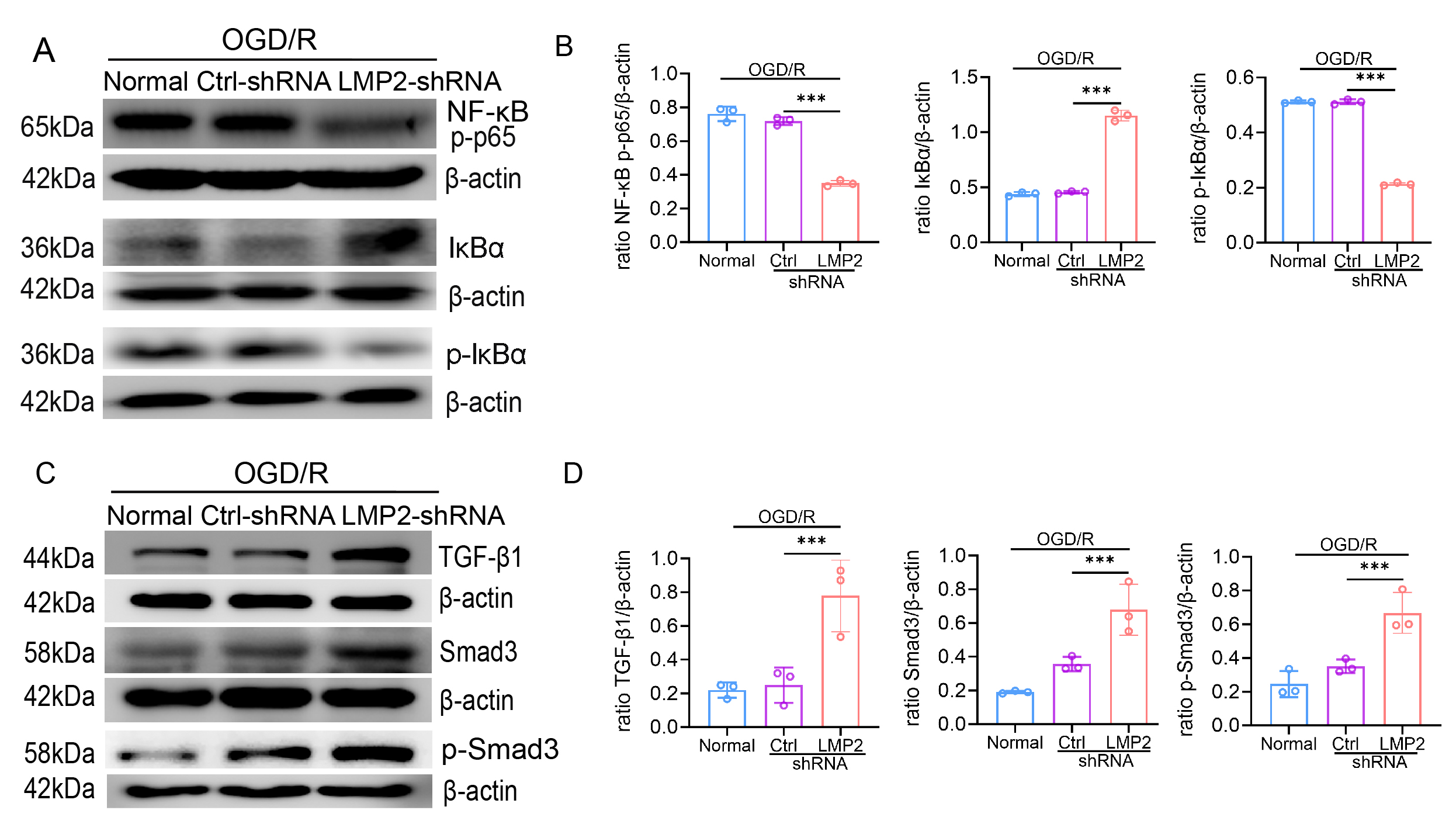
**

**Supplementary Figure 10. Effects of LMP2 knockdown on NF-κB and TGF-β1/Smad3 signaling pathways in CTX-TNA2 astrocytes under OGD/R conditions.**(A-B) Representative Western blot images and quantification of p-NF-κB p65, IκBα, and p-IκBα protein expression in CTX-TNA2 astrocytes under Normal, Control-shRNA, and LMP2-shRNA conditions following OGD/R. (C-D) Representative Western blot images and quantification of TGF-β1, Smad3, and p-Smad3 protein expression in CTX-TNA2 astrocytes under the indicated conditions.The Normal group represents non-transfected cells subjected to OGD/R. Data are presented as mean ± SD from three independent experiments using one-way ANOVA with LSD’s post hoc test. ^***^*P* < 0.001.


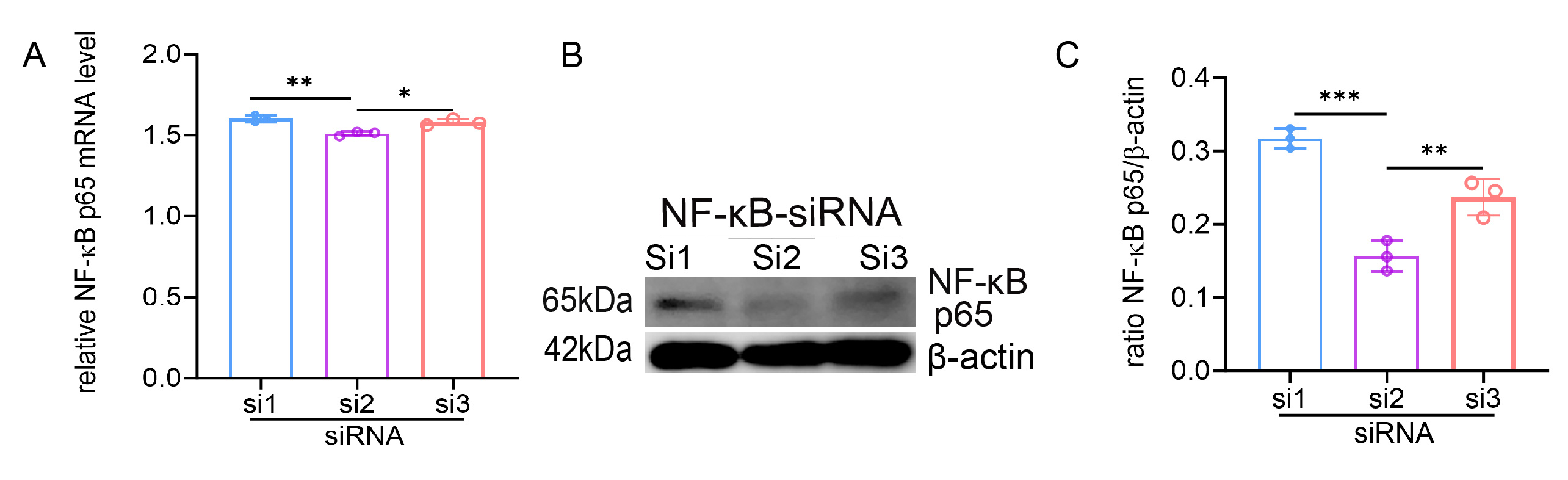


**Supplementary Figure 11. Screening and validation of NF-κB p65 siRNA sequences in CTX-TNA2 astrocytes.** (A) Relative NF-κB p65 mRNA expression in CTX-TNA2 astrocytes following transfection with three independent NF-κB p65 siRNA sequences (si1-si3), as determined by RT-qPCR. (B-C) Representative Western blot images and quantification of NF-κB p65 protein expression following transfection with the indicated siRNA sequences. Data are presented as mean ± SD from three independent experiments using one-way ANOVA with LSD’s post hoc test. ^*^*P* < 0.05, ^**^*P* < 0.01, ^***^*P* < 0.001.


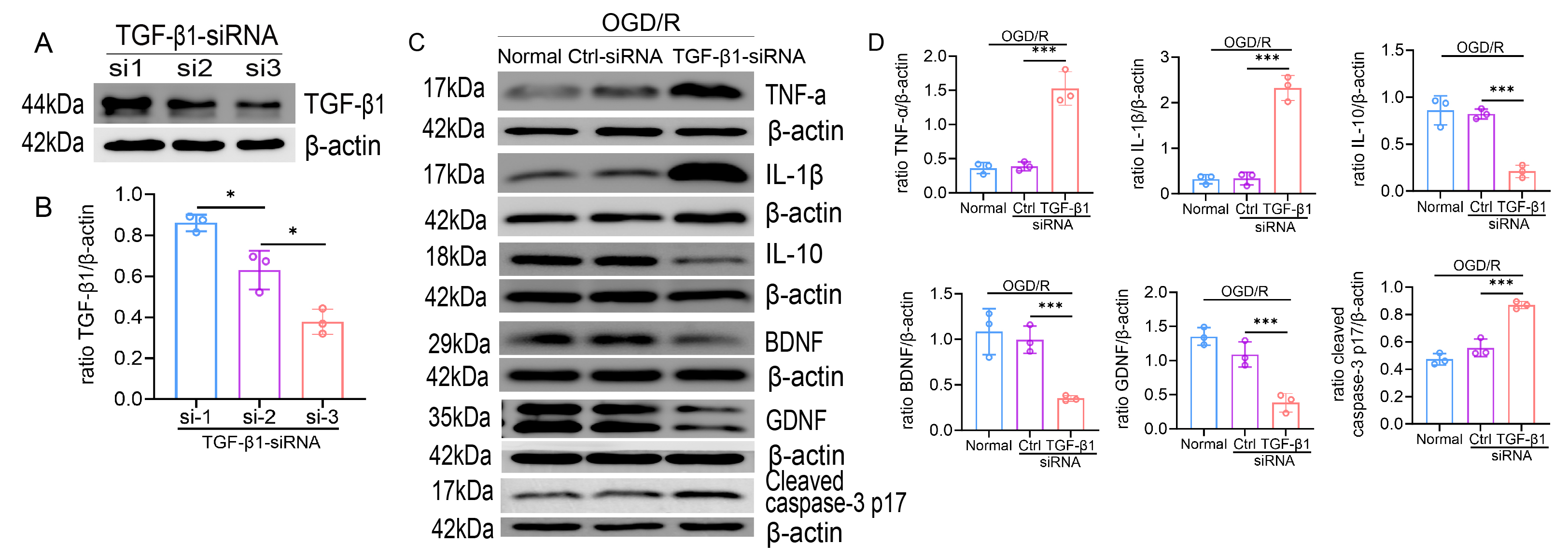


**Supplementary Figure 12. Effects of TGF-β1 knockdown on inflammatory, neurotrophic, and apoptosis-related proteins in CTX-TNA2 astrocytes under OGD/R conditions. (A-B) Representative Western blot images and quantification of TGF-β1 protein expression in CTX-TNA2 astrocytes following transfection with three independent TGF-β1 siRNA sequences (si1-si3). (C-D) Representative Western blot images and quantification of TNF-α, IL-1β, IL-10, BDNF, GDNF, and cleaved caspase-3 p17 protein expression in CTX-TNA2 astrocytes under OGD/R conditions. Normal represents non-transfected CTX-TNA2 astrocytes subjected to OGD/R, whereas Ctrl-siRNA and TGF-β1-siRNA represent OGD/R-treated cells transfected with control siRNA or TGF-β1 siRNA, respectively.** Data are presented as mean ± SD from three independent experiments using one-way ANOVA with LSD’s post hoc test. ^*^*P* < 0.05, ^**^*P* < 0.01, ^***^*P* < 0.001.
