# Supplementary material for: Astrocytic LMP2 Coordinates NF‐κB and TGF‐β1/Smad3 Signaling to Drive Neuroinflammation after Cerebral Ischemia/Reperfusion
Source: Adv Sci (Weinh). 2026 Jul 27:e23902. Online ahead of print. doi: 10.1002/advs.202523902 (PMC13403376; doi:10.1002/advs.202523902)
Supplement: Supplementary file 2 — Supporting File 2: advs76807‐sup‐0002‐TablesS1.docx. [file ADVS-9999-e23902-s002.docx]

Table 1. Primary and secondary antibodies for immunohistochemistry or immunocytochemistry

| **Antibody** | **Brand** | **Catalog No.** | **Dilution** |
| --- | --- | --- | --- |
| Mouse anti-GFAP (2E1) | Santa Cruz Biotechnology | sc-33673 | 1:100 |
| Anti-C3 antibody (EPR19394) | Abcam | ab200999 | 1:100 |
| S100A10 Polyclonal Antibody | Invitrogen | PA5-95505 | 1:100 |
| Vimentin (D21H3) XP® Rabbit mAb | Cell Signaling Technology | #5741 | 1:100 |
| NF-κB p65 (D14E12) XP® Rabbit mAb | Cell Signaling Technology | #8242 | 1:100 |
| NeuN Rabbit pAb | ABclonal | A0951 | 1:100 |
| Anti-Iba1 antibody (rabbit; microglial marker) | Abcam | ab178846 | 1:100 |
| Anti-proteasome 20S LMP2 antibody (rabbit) | Abcam | ab184172 | 1:100 |
| Anti-CD31 antibody (mouse) | Abcam | ab24590 | 1:100 |
| Phospho-Smad3 (Ser425) antibody (rabbit) | Affinity Biosciences | AF3362 | 1:100 |
| TGF-β1 antibody (rabbit polyclonal) | Affinity Biosciences | AF1027 | 1:100 |
| Alexa Fluor® 488-conjugated goat anti-mouse IgG (H+L), F(ab')2 Fragment | Cell Signaling Technology | #4408 | 1:1000 |
| Alexa Fluor® 594-conjugated goat anti-mouse IgG (H+L), F(ab')2 Fragment | Cell Signaling Technology | #8890 | 1:1000 |
| Alexa Fluor® 488-conjugated goat anti-rabbit IgG (H+L), F(ab')2 Fragment | Cell Signaling Technology | #4412 | 1:1000 |
| Alexa Fluor® 594-conjugated goat anti-rabbit IgG (H+L), F(ab')2 Fragment | Cell Signaling Technology | #8889 | 1:1000 |

Table 2. Primary and secondary antibodies for Western blot

| **Antibody** | **Brand** | **Catalog No.** | **Dilution** |
| --- | --- | --- | --- |
| Anti-proteasome 20S LMP2 antibody (rabbit) | Abcam | ab184172 | 1:3000 |
| Mouse anti-IL-1β | Abcam | ab2105 | 1:500 |
| Mouse anti-TNF-α | Abcam | ab205587 | 1:500 |
| CXCL10 rabbit polyclonal antibody | Affinity Biosciences | DF6417 | 1:500 |
| MMP9 rabbit polyclonal antibody | Affinity Biosciences | AF5228 | 1:500 |
| BAX rabbit polyclonal antibody | Affinity Biosciences | AF0120 | 1:1000 |
| BCL2 rabbit polyclonal antibody | Affinity Biosciences | AF6139 | 1:1000 |
| Cleaved-caspase-3 (Asp175), p17 antibody | Affinity Biosciences | AF7022 | 1:500 |
| GDNF antibody | Invitrogen | PA5-89957 | 1:1000 |
| Anti-BDNF antibody [3B2] | Abcam | ab205067 | 1:1000 |
| IL-10 monoclonal antibody | Invitrogen | JES3-9D7 | 1:500 |
| β-Actin (8H10D10) Mouse mAb | Cell Signaling Technology | #3700 | 1:5000 |
| Anti-C3 antibody (EPR19394) | Abcam | ab200999 | 1:1000 |
| C3 antibody | Affinity Biosciences | DF13224 | 1:1000 |
| S100A10 polyclonal antibody | Proteintech | 11250-1-AP | 1:1000 |
| NF-κB p65 (D14E12) XP® Rabbit mAb | Cell Signaling Technology | #8242 | 1:1000 |
| Phospho-NF-κB p65 (Ser536) (93H1) Rabbit mAb | Cell Signaling Technology | #3033 | 1:1000 |
| Smad3 antibody | Affinity Biosciences | AF6362 | 1:1000 |
| Phospho-Smad3 (Ser425) antibody (rabbit) | Affinity Biosciences | AF3362 | 1:1000 |
| LMP7 antibody (D-2) mouse mAb | Santa Cruz Biotechnology | sc-374089 | 1:1000 |
| PSMB10/MECL-1 polyclonal antibody (rabbit) | Invitrogen | PA5-96327 | 1:1000 |
| PSMB6 polyclonal antibody | Proteintech | 11684-2-AP | 1:1000 |
| IκBα polyclonal antibody | Proteintech | 10268-1-AP | 1:1000 |
| Phospho-IκBα (Ser32/36) recombinant monoclonal antibody | Proteintech | 82349-1-RR | 1:1000 |
| Lamin B1 polyclonal antibody | Proteintech | 12987-1-AP | 1:1000 |
| GFAP (E4L7M) XP® Rabbit mAb | Cell Signaling Technology | #80788 | 1:1000 |
| TGF-β1 antibody (rabbit) | Affinity Biosciences | AF1027 | 1:1000 |
| GAPDH monoclonal antibody | Proteintech | 60004-1-Ig | 1:1000 |
| Anti-mouse IgG, HRP-linked antibody | Cell Signaling Technology | #7076 | 1:7000 |
| Anti-rabbit IgG, HRP-linked antibody | Cell Signaling Technology | #7074 | 1:5000 |
